# Holographic-(V)AE: An end-to-end SO(3)-equivariant (variational) autoencoder in Fourier space

**DOI:** 10.1103/physrevresearch.6.023006

**Published:** 2024-04-01

**Authors:** Gian Marco Visani, Michael N. Pun, Arman Angaji, Armita Nourmohammad

**Affiliations:** Paul G. Allen School of Computer Science and Engineering, University of Washington, 85 E Stevens Way NE, Seattle, Washington 98195, USA; Department of Physics, University of Washington, 3910 15th Avenue Northeast, Seattle, Washington 98195, USA; Institute for Biological Physics, University of Cologne, Zülpicher Str. 77, 50937 Cologne, Germany; Department of Physics, University of Washington, 3910 15th Avenue Northeast, Seattle, Washington 98195, USA;; Paul G. Allen School of Computer Science and Engineering, University of Washington, 85 E Stevens Way NE, Seattle, Washington 98195, USA;; Department of Applied Mathematics, University of Washington, 4182 W Stevens Way NE, Seattle, Washington 98105, USA; and Fred Hutchinson Cancer Center, 1241 Eastlake Ave E, Seattle, Washington 98102, USA

## Abstract

Group-equivariant neural networks have emerged as an efficient approach to model complex data, using generalized convolutions that respect the relevant symmetries of a system. These techniques have made advances in both the supervised learning tasks for classification and regression, and the unsupervised tasks to generate new data. However, little work has been done in leveraging the symmetry-aware expressive representations that could be extracted from these approaches. Here, we present *holographic*-(variational) autoencoder [H-(V)AE], a fully end-to-end SO(3)-equivariant (variational) autoencoder in Fourier space, suitable for unsupervised learning and generation of data distributed around a specified origin in 3D. H-(V)AE is trained to reconstruct the spherical Fourier encoding of data, learning in the process a low-dimensional representation of the data (i.e., a latent space) with a maximally informative rotationally invariant embedding alongside an equivariant frame describing the orientation of the data. We extensively test the performance of H-(V)AE on diverse datasets. We show that the learned latent space efficiently encodes the categorical features of spherical images. Moreover, the low-dimensional representations learned by H-VAE can be used for downstream data-scarce tasks. Specifically, we show that H-(V)AE’s latent space can be used to extract compact embeddings for protein structure microenvironments, and when paired with a random forest regressor, it enables state-of-the-art predictions of protein-ligand binding affinity.

## I. INTRODUCTION

In supervised learning, e.g., for classification tasks, the success of state-of-the-art algorithms is often attributed to respecting known inductive biases of the function they are trying to approximate. One such bias is the invariance of the function to certain transformations of the input. For example, image classification should be translationally invariant, in that the output should not depend on the position of the object in the image. To achieve such invariance, conventional techniques use data augmentation to train an algorithm on many transformed forms of the data. However, this solution is only approximate and increases training time significantly, up to prohibitive scales for high-dimensional and continuous transformations (~500 augmentations are required to learn 3D rotation-invariant patterns [1]). Alternatively, one could use invariant features of the data (e.g., pairwise distance between different features) as input to train any machine learning algorithm [2]. However, the choice of these invariants is arbitrary and the resulting network could lack in expressiveness.

Recent advances have brought concepts from group theory to develop symmetry-aware neural network architectures that are equivariant under actions of different symmetry groups [3–12]. Equivariance with respect to a symmetry group is the property that, if the input is transformed via a group action, then the output is transformed according to a linear operation determined by the symmetry group itself; it is easy to see that invariance is a special case of equivariance, where the linear operation is simply the identity. These group equivariant networks can systematically treat and interpret various transformation in data, and learn models that are agnostic to the specified transformations. For example, models equivariant to euclidean transformations have recently advanced the state-of-the-art on many supervised tasks for classification and regression [3–12]. These models are more flexible and data efficient compared to their purely invariant counterparts [1,13].

Extending such group-invariant and equivariant paradigms to unsupervised learning (i.e., for modeling the data distribution) could map out compact representations of data that are agnostic to a specified symmetry transformation. In machine learning, autoencoders (AE’s) and their probabilistic version, variational autoencoders (VAE’s), are among the artificial neural networks that are commonly used for unsupervised learning, in that they provide an efficient representation of unlabeled data [14]. However, little work has been done to implement group equivariance in autoencoder architectures [15]. Recently, generative models based on normalizing flows [11] and diffusion models [16–18] that are equivariant to subsets of euclidean transformations have been applied to the task of molecule and protein generation in 3D. Although powerful in generative tasks, these classes of models do not learn compact representations of the data, as autoencoders do. The key benefit of autoencoder generative models is that their learned compact representations can be used for downstream tasks in semisupervised learning algorithms (as we show in this paper), and can help identify relevant and semantically meaningful features and patterns from high-dimensional complex data, projected down into lower-dimensional representations [19].

Here, we focus on developing neural network architectures—and in particular (variational) autoencoders—for unsupervised learning that are equivariant to rotations around a specified origin in 3D, denoted by the group SO(3). To define rotationally equivariant transformations, it is convenient to project data to spherical Fourier space [6]. Accordingly, we encode the data in spherical Fourier space by constructing *holograms* of the data that are conveniently structured for equivariant operations. These data holograms are inputs to our SO(3)-equivariant (variational) autoencoder in spherical Fourier space, with a fully equivariant encoder-decoder architecture trained to reconstruct the Fourier coefficients of the input; we term this approach *holographic*-(V)AE [or H-(V)AE]. Our network learns an SO(3)-equivariant latent space. Notably, the latent space of H-(V)AE disentangles the invariant and equivariant features of data, which describe its semantics and orientation, respectively. This algorithmic property is similar to that of the recent approaches in unsupervised learning of disentangled representations, shown to be powerful for image generation and style mixing at different scales [20,21].

We extensively test the performance and properties of H-(V)AE on two domains. First, we focus on spherical images, demonstrating high accuracy in unsupervised classification and clustering tasks. Second, we focus on structural biology, and demonstrate that H-(V)AE can be effectively used to construct compact, informative, and symmetry-aware representations of protein structures, which can be used for downstream tasks. Specifically, we leverage H-(V)AE trained on a large corpus of protein structure microenvironments to construct local representations of protein-ligand binding pockets that are both rotationally and translationally equivariant [i.e., SE(3) equivariant]. When combined with a simple random forest regressor, we achieve state-of-the-art accuracy on the task of predicting the binding affinity between a protein and a ligand in complex. Our code and pretrained models are available on GitHub [22].

## II. MODEL

### Representation and transformation of 3D data in spherical bases

We are interested in modeling 3D data (i.e., functions in $R^{3}$), for which the global orientation of the data should not impact the inferred model [23]. We consider functions distributed around a specified origin, which we express by the resulting spherical coordinates (r,θ,ϕ) around the origin; θ and ϕ are the azimuthal and the polar angles and r defines the distance to the reference point in the spherical coordinate system. In this case, the set of rotations about the origin define the 3D rotation group SO(3), and we will consider models that are rotationally equivariant under SO(3).

To define rotationally equivariant transformations, it is convenient to project data to spherical Fourier space. We use spherical harmonics to encode the angular information of the data. Spherical harmonics are a class of functions that form a complete and orthonormal basis for functions f(θ,ϕ) defined on a unit sphere (r=1). In their complex form, spherical harmonics are defined as

(1) $$Y_{\ell m}\left(\theta ,\phi \right)=\sqrt{\frac{2n+1}{4\pi }\frac{\left(n-m\right)!}{\left(n+m\right)!}}e^{im\phi }P_{\ell }^{m}\left(\text{cos}\;\theta \right),$$

where ℓ is a non-negative integer (0⩽ℓ) and m is an integer within the interval $-\ell ⩽m⩽\ell .P_{\ell }^{m}(cos\theta )$ is the Legendre polynomial of degree ℓ and order m, which, together with the complex exponential $e^{im\phi }$, define sinusoidal functions over the angles θ and ϕ. In quantum mechanics, spherical harmonics are used to represent the orbital angular momenta, e.g., for an electron in a hydrogen atom. In this context, the degree ℓ relates to the eigenvalue of the square of the angular momentum, and the order m is the eigenvalue of the angular momentum about the azimuthal axis.

To encode a general function ρ(r,θ,ϕ) with both radial and angular components, we use the Zernike Fourier transform,

(2) $${\hat{Z}}_{\ell m}^{n}=\int \rho \left(r,\theta ,\phi \right)Y_{\ell m}\left(\theta ,\phi \right)R_{\ell }^{n}\left(r\right)dV,$$

where $Y_{\ell m}(\theta ,\phi )$ is the spherical harmonics of degree ℓ and order m, and $R_{\ell }^{n}(r)$ is the radial Zernike polynomial in 3D (Eq. (A7)) with radial frequency n⩾0 and degree ℓ. $R_{\ell }^{n}(r)$ is nonzero only for even values of n-ℓ⩾0. Zernike polynomials form a complete orthonormal basis in 3D, and therefore, can be used to expand and retrieve 3D shapes, if large enough ℓ and n values are used; approximations that restrict the series to finite n and ℓ are often sufficient for shape retrieval, and hence, desirable algorithmically. Thus, in practice, we cap the resolution of the ZFT to a maximum degree L and a maximum radial frequency N.

A class of functions that we consider in this paper are 3D point clouds, e.g., the atomic composition of a protein in space. We represent point clouds ρ(r)≡ρ(r,θ,ϕ) by the sum of Dirac-δ functions centered at each point,

(3) $$\rho \left(r\right)=\sum_{i\in \text{points}}\delta \left(\rho \left(r_{i}\right)-\rho \left(r\right)\right),$$

where δ(x)=1 for x=0 and it is zero, otherwise. The resulting ZFT of a point cloud follows a closed form, and notably, it does not require a discretization of 3D space for numerical computation,

(4) $${\hat{Z}}_{\ell m}^{n}=\sum_{i\in \text{points}}R_{n}^{\ell }\left(r_{i}\right)Y_{\ell m}\left(\theta_{i},\varphi_{i}\right).$$

We can reconstruct the data using the inverse ZFT and define approximations by truncating the angular and radial frequencies at L and N (see Sec. A1).

Conveniently, the angular representation of the data by spherical harmonics in a ZFT transform forms an equivariant basis under rotation in 3D, implying that if the input (i.e., atomic coordinates of a protein) is rotated, then the output is transformed according to a linear operation determined by the rotation. The linear operator that describes how spherical harmonics transform under rotations are called the Wigner D-matrices. Notably, Wigner D-matrices are the irreducible representations (irreps) of SO(3). Therefore, the SO(3) group acts on spherical Fourier space via a direct sum of irreps. Specifically, the ZFT encodes a data point into a *tensor* composed of a direct sum of *features*, each associated with a degree ℓ indicating the irrep that it transforms with under the action of SO(3). We refer to these tensors as SO(3)-*steerable tensors* and to the vector spaces they occupy as SO(3)-*steerable vector spaces*, or simply *steerable* for short since we only deal with the SO(3) group in this paper.

**FIG. 1.**
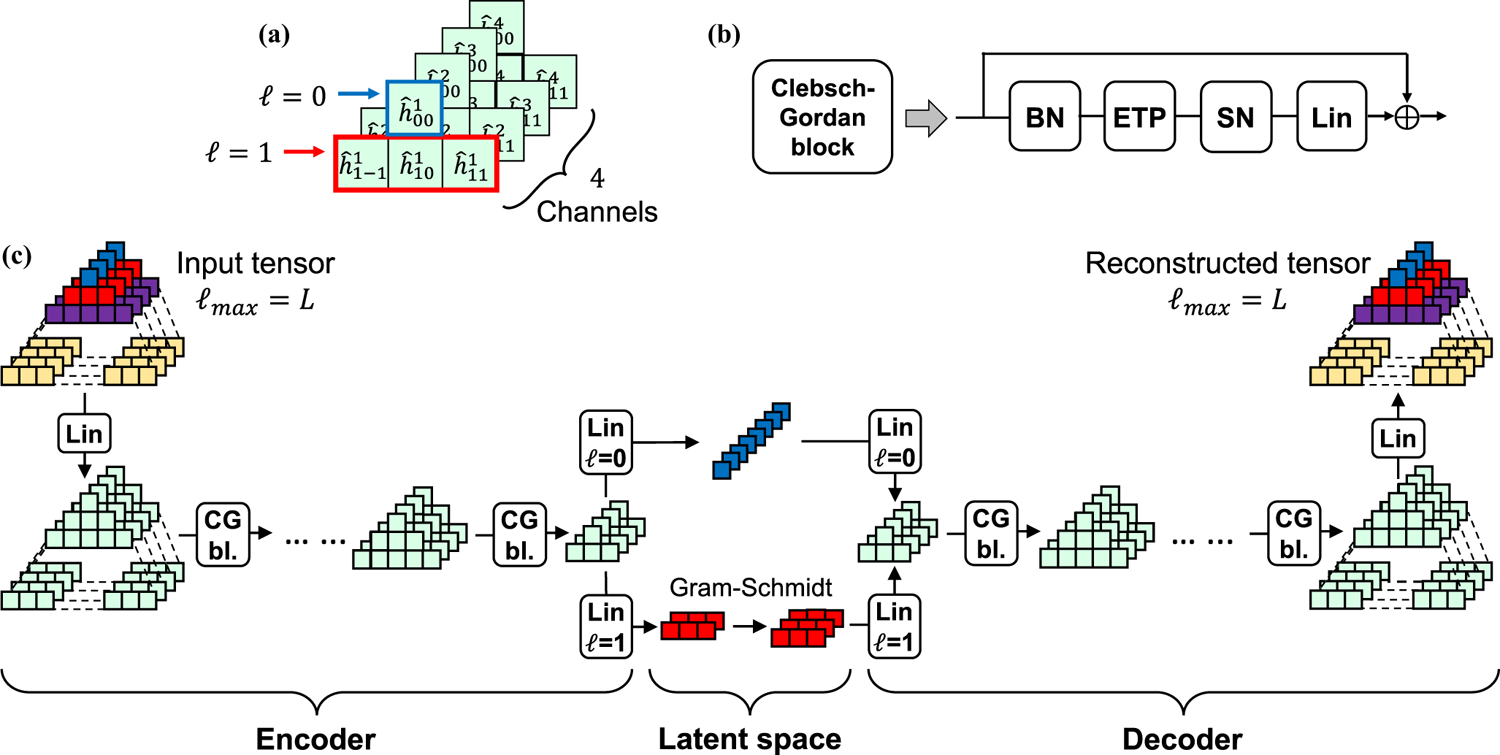
Schematic of the network architecture. (a) Schematic of a steerable tensor with $\ell_{max}=1$ and 4 channels per feature degree. We choose a pyramidal representation that naturally follows the expansion in size of features of higher degree. (b) Schematic of a Clebsch-Gordan block (CG bl.), with batch norm (BN), efficient tensor product (ETP), and signal norm (SN), and linear (Lin) operations. (c) Schematic of the H-AE architecture. We color code features of different degrees in the input and in the latent space for clarity. The H-VAE schematic differs only in the latent space, where two sets of invariants are learned (means and standard deviations of an isotropic Gaussian distribution).

We note that a tensor may contain multiple features of the same degree ℓ, which we generically refer to as distinct *channels* c. For example, for 3D atomic point clouds, these features include the identity and chemical properties of the constituent atoms. Throughout the paper, we refer to generic steerable tensors as h and index them by ℓ (degree of $Y_{\ell m}$), m (order of $Y_{\ell m}$) and c (channel type). We adopt the “hat” notation for individual entries (e.g., ${\overset{ˆ}{h}}_{\ell m}$) to remind ourselves of the analogy with Fourier coefficients; see Fig. 1(a) for a graphical illustration of a tensor.

One key feature of neural networks is applying nonlinear activations, which enable a network to approximately model complex and nonlinear phenomena. Commonly used nonlinearities include reLU, tanh, and softmax functions. However, these conventional nonlinearities can break rotational equivariance in the Fourier space. To construct expressive rotationally equivariant neural networks we can use the Clebsch-Gordan (CG) tensor product ${⊗}_{cg}$, which is the natural nonlinear (more specifically, bilinear in the case of using two sets of Fourier coefficients) operation in the space of spherical harmonics [24].

The CG tensor product combines two features of degrees $\ell_{1}$ and $\ell_{2}$ to produce another feature of degree $\left|\ell_{2}-\ell_{1}\right|⩽\ell_{3}⩽\left|\ell_{1}+\ell_{2}\right|$. Let $h_{\ell }\in R^{2\ell +1}$ be a generic degree ℓ tensor, with individual components ${\overset{ˆ}{h}}_{\ell m}$ for -ℓ⩽m⩽ℓ. The CG tensor product is given by

(5) $${\hat{h}}_{\ell_{3}m_{3}}={\left(h_{\ell_{1}}{⊗}_{cg}h_{\ell_{2}}\right)}_{\ell_{3}m_{3}}\;=\overset{\ell_{1}}{\underset{m_{1}=-\ell_{1}}{\sum }}\overset{\ell_{2}}{\underset{m_{2}=-\ell_{2}}{\sum }}C_{\left(\ell_{1}m_{1}\right)\left(\ell_{2}m_{2}\right)}^{\left(\ell_{3}m_{3}\right)}{\hat{h}}_{\ell_{1}m_{1}}{\hat{h}}_{\ell_{2}m_{2}},$$

where $C_{\left(\ell_{1}m_{1}\right)\left(\ell_{2}m_{2}\right)}^{\left(\ell_{3}m_{3}\right)}$ are the Clebsch-Gordan coefficients, and can be precomputed for all degrees of spherical tensors [24]. Similar to spherical harmonics, Clebsch-Gordan tensor products also appear in quantum mechanics, and they are used to express couplings between angular momenta. In following with recent study on group-equivariant machine learning [6], we will use Clebsch-Gordan products to express nonlinearities in 3D rotationally equivariant neural networks for protein structures.

## III. HOLOGRAPHIC-(V)AE (H-(V)AE)

### Network architecture and training.

H-(V)AE consists of an encoder that, through learned linear projections and preset nonlinear operations, project the data onto a compressed rotationally equivariant latent space. A trained decoder that is similarly constructed then takes this latent projection and reconstructs the input data. The combination of leaned linear and preset nonlinear operations form equivariant Clebsch-Gordan blocks (CG bl.) both for the encoder and the decoder; see Fig. 1 and below for details on the structure of a Clebsch-Gordan block.

Using the Clebsch-Gordan blocks, we construct a fully rotationally equivariant architecture for unsupervised learning. Specifically, the encoder—denoted by the function $E_{\phi }$—takes as input a steerable tensor with maximum degree $\ell_{max}=L$ and, via a stack of Clebsch-Gordan blocks, iteratively and equivariantly transfers information from higher degrees to lower ones, down to the final encoder layer with $\ell_{max}=1$, resulting in the invariant (ℓ=0) and the frame-defining equivariant (ℓ=1) embeddings. The frame is constructed by learning two vectors from the ℓ=1 embedding in the final layer of the encoder and using Gram-Schmidt to find the corresponding orthonormal basis [25]. The third orthonormal basis vector is then calculated as the cross product of the first two.

The decoder—denoted by the function $D_{\theta }$—learns to reconstruct the input from the invariant (ℓ=0) embedding of the encoder’s final layer z and the frame g, iteratively increasing the maximum degree $\ell_{\text{max}}$ of the intermediate representations by leveraging the CG tensor product within the Clebsch-Gordan blocks [Fig. 1(c)]. We refer the reader to Sec. A2e for further details on the design choices of the network.

To add stochasticity and make the model variational (i.e., constructing H-VAE as opposed to H-AE), we parametrize the *invariant* part of the latent space by an isotropic Gaussian, i.e., we learn the parameters of the Gaussian posterior distribution $q_{\phi }(z|x)$, which is trained to match a prior p(z). Thus, a trained H-VAE can be used to generate samples $x^{\prime }$ from the data distribution by first sampling from the latent space according to z~p(z), and then feeding z into the decoder alongside a frame g of choice, $x^{\prime }=D_{\theta }(z,g)$.

We train H-(V)AE to minimize the reconstruction loss ${ℒ}_{rec}$ between the input and the reconstructed tensors. Note that for each input tensor x the reconnected tensor $x^{\prime }$ is generated by first using the encoder to map the input onto an invariant embedding z and a frame g [i.e., $\left(z,g)=E_{\phi }(x\right)$] and then using the decoder to reconstruct [i.e., $x^{\prime }=D_{\theta }(z,g)$]; both the encoder and the decoder are trained by minimizing the reconstruction loss ${ℒ}_{\text{rec}}$. For H-VAE only, we we further minimize the Kullback-Leibler divergence $D_{KL}$ of the posterior invariant latent space distribution $q_{\phi }(z|x)$ from the selected prior p(z) [isotropic normal in this paper, p(z)=𝒩(0,I)] [14], resulting in the complete loss function $ℒ\left(x,x^{\prime }\right)$,

(6) $$ℒ\left(x,x^{\prime }\right)=\alpha {ℒ}_{\text{rec}}\left(x,x^{\prime }\right)+\beta D_{\text{KL}}\left(q_{\phi }\left(z|x\right)∥p\left(z\right)\right).$$

For H-VAE, the encoder $E_{\phi }$ is a differentiable reparameterization of the posterior distribution $q_{\phi }(z|x)$. Specifically, $E_{\phi }$ additionally takes noise ε~𝒩(0,I) as input, so that the invariant latent embedding z outputted from $E_{\phi }(x,\varepsilon )$ is regarded as a sample from $q_{\phi }(z|x)$. This “reparameterization” trick allows for error propagation through the Gaussian latent variables, and is essential for VAE training [14].

We use mean square error (MSE) for ${ℒ}_{\text{rec}}$, which as we show in Sec. A2h, respects the necessary property of SO(3)-pairwise invariance, ensuring that the model remains rotationally equivariant. Hyperparameters α and β control the trade-off between reconstruction accuracy and latent space regularization [26]; see Sec. A2f for details on tuning of these rates during training.

As a result of this training, H-(V)AE learns a *disentangled* latent space consisting of a maximally informative invariant (ℓ=0) component z of arbitrary size, as well as three orthonormal vectors (ℓ=1), which represent the global 3D orientation of the object and reflect the *coordinate frame* of the input tensor. Crucially, the disentangled nature of the latent space is respected at all stages of training, and is guaranteed by the model’s rotational equivariance. We empirically verify the equivariance of our model up to numerical errors in Table S1 within the Supplemental Material (SM) [28].

### Invariant conditioning.

Optionally, H-VAE can be made to model data distributions *conditioned* on some variable c. A conditional H-VAE (H-CVAE) can be used to sample data from a conditional distribution x~p(x∣c) (e.g., sampling handwritten digits conditioned on the digit identity) [29]. Furthermore, conditioning makes it so that the latent representation learned by H-CVAE is devoid of information pertaining to the conditioning variable (e.g., a latent representation of handwritten digits from a model conditioned on the digit identity would not contain information about the digit identity). In practice, conditioning is applied by simply adding c as input to both the encoder and the decoder. In our experiments, we only condition on the *invariant* variables by adding them as ℓ=0 features.

### Architecture of a Clebsch-Gordan block.

Each Clebsch-Gordan block (CG bl.) consists of a trained linear layer [*linearity* (Lin)], an efficient tensor product (ETP) to inject nonlinearity in the network, and normalization steps by [*batch norm* (BN)] and [*signal norm* (SN)] to respectively speed-up convergence and stabilize training.

### Linearity (Lin).

A linear layer acts on steerable tensors by learning degree-specific linear operations. Linear layers are trained in that we learn weight matrices specific to each degree ℓ, and use them to map across degree-ℓ feature spaces by learning linear combinations of degree-ℓ features in the input tensor. Specifically, let us consider a vector $h_{\ell }$, containing features of the same degree ℓ. We train the network to learn the weight matrix $W_{\ell }$ to map $h_{\ell }$ to $h_{\ell }^{\prime }$ between the network’s layers, i.e., $h_{\ell }^{\prime }=W_{\ell }h_{\ell }$ (see Sec. A2a for details).

### Nonlinearity with efficient tensor product (ETP).

One key feature of neural networks is applying nonlinear activations, which enable a network to approximately model complex and nonlinear phenomena. As noted above, we use the Clebsch-Gordan tensor product to inject rotationally equivariant nonlinearities in the network. Specifically, within a Clebsch-Gordan block the output of a linear layer is acted upon by bilinear CG tensor product, as was originally prescribed by in Ref. [6] for SO(3)-equivariant convolutional neural networks. This bilinear operation enables information flow between features of different degrees, which is necessary for constructing expressive models, and for transferring higher-ℓ information to ℓ=0 in H-(V)AE ’s invariant encoder, and back in the decoder.

To significantly reduce the computational and memory costs of the tensor products, we perform *efficient tensor products* (ETPs) by leveraging some of the modifications proposed in Ref. [30]. Specifically, we compute tensor products channel-wise, i.e., only between features belonging to the same channel, and we limit the connections between features of different degrees. We found these modifications to be necessary to efficiently work with data encoded in large number of channels C and with large maximum degree L; see Sec. A2b for details, and Table S2 within the SM [28] for an ablation study showing the improvement in parameter efficiency provided by the ETP.

### Batch and signal norm.

We normalize intermediate tensor representations degree-wise and channel-wise by the batch-averaged norms of the features, as initially proposed in Ref. [6]; see Fig. 1(b), Sec. A2c, and Fig. S1 within the SM [28] for details. We found using batch norm alone often caused activations to explode in the decoder during evaluation. Thus, we introduce signal norm, whereby we divide each steerable tensor by the square root of its *total* norm, defined as the sum of the norms of each of the tensor’s features, and apply a degree-specific affine transformation for added flexibility; see Sec. A2d for mathematical details. Signal norm can be seen as a form of the classic layer normalization that respects SO(3) equivariance [31].

## IV. RESULTS

### A. Rotated MNIST on the sphere

We extensively test the performance of H-(V)AE on the MNIST-on-the-sphere dataset [32]. Following Ref. [4], we project the MNIST dataset, which includes images of handwritten numbers, onto the lower hemisphere of a discrete unit sphere. We consider two variants of training/test set splits, NR/R and R/R, differing in whether the training/test images have been randomly rotated (R) or not (NR). For each dataset, we map the images to steerable tensors via the Zernike Fourier transform (ZFT) in Eq. (2) and train models with different different sizes of latent spaces (z=16 and z=120) and model types (AE and VAE). In all cases the model architecture follows from Fig. 1(c); see Sec. A5b for details.

For variational models, we tune the regularization strength β to maximize the expected quality of the generated samples. We define samples to be of high quality if (i) they can be correctly classified by a classifier trained on real data, and (ii) they are diverse enough, indicating that the model is not overfitting; we found that there is a trade-off between classification accuracy and the variability in the generated samples (Fig. S4 within the SM [28]). We leverage H-CVAE models conditioned with digit identity to generate digit-specific images associated with each value of β. See Sec. A5b for more details. Using our procedure we select values of β=0.6 and β=2.0 for H-VAE models with latent space sizes of z=16 and z=120, respectively.

We use the metric *cosine loss* to measure a model’s reconstruction ability. Cosine loss is a normalized dot product generalized to operate on pairs of steerable tensors (akin to cosine similarity), and modified to be interpreted as a loss. Importantly, unlike MSE, cosine loss is dimensionless, and therefore, comparable across different datasets and encodings of data in tensors of different sizes (network hyperparameters), though it is agnostic to magnitudes and thus unfit for training; see Sec. A2j for details.

**FIG. 2.**
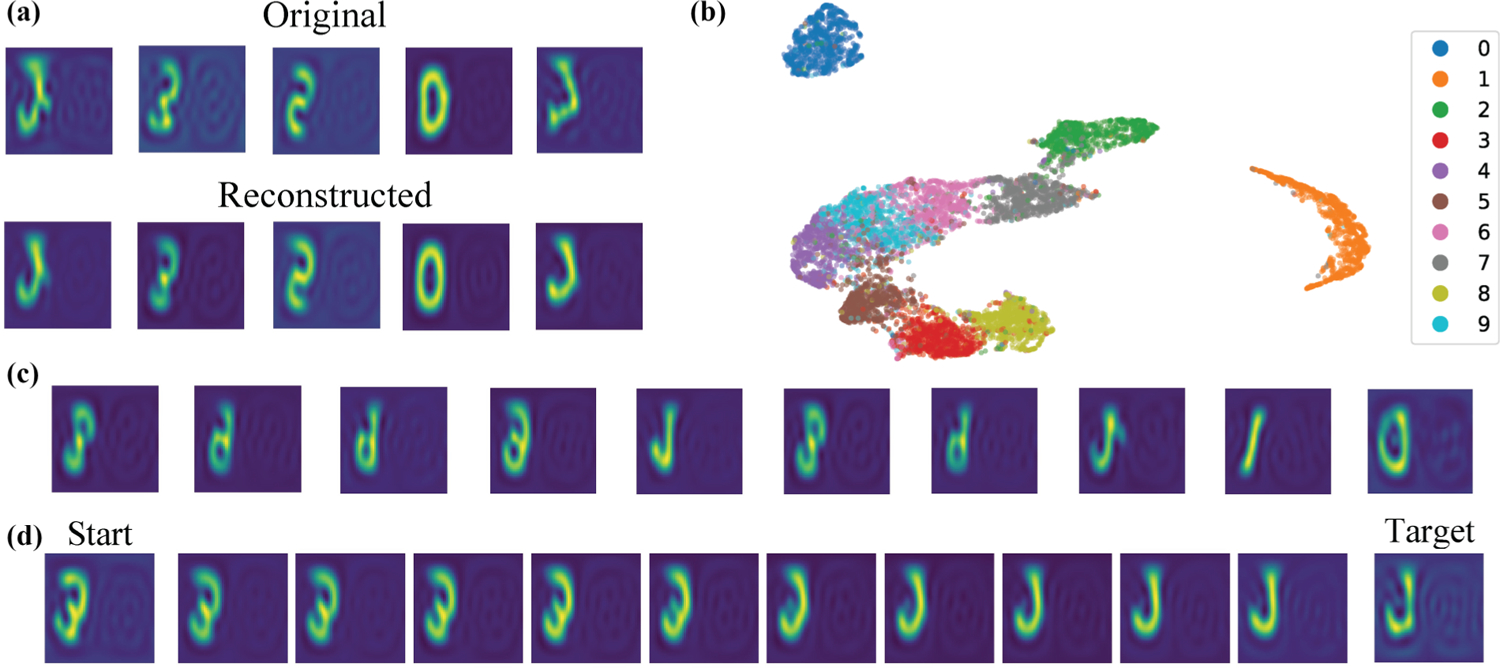
H-VAE on MNIST-on-the-sphere. Evaluation on rotated digits for an H-VAE trained on nonrotated digits with z=16. (a) Original and reconstructed images in the canonical frame after inverse transform from Fourier space. The images are projected onto a plane. Distortions at the edges and flipping are side-effects of the projection. (b) Visualization of the latent space via 2D UMAP [27]. Data points are colored by digit identity. (c) Cherry-picked images generated by feeding the decoder invariant embeddings sampled from the prior distribution and the canonical frame. (d) Example image trajectory by linearly interpolating through the learned invariant latent space. Interpolated invariant embeddings are fed to the decoder alongside the canonical frame. MNIST-on-the-sphere dataset is created by projecting data from the planar MNIST on a discrete unit sphere, using the Driscoll-Healey (DH) method with a bandwidth (bw) of 30 [4].

All trained models achieve very low reconstruction cosine loss (Table I) with no significant difference between training modes, indicating that the models successfully leverage SO(3) equivariance to generalize to unseen orientations. Predictably, AE models have lower reconstruction loss than VAE models [since they do not need to find a trade-off between reconstruction error and KL divergence, Eq. (6)], and so do models with a larger latent space. Nonetheless, H-VAE achieves reliable reconstructions, as shown in Fig. 2(a) and Table I.

**FIG. 3.**
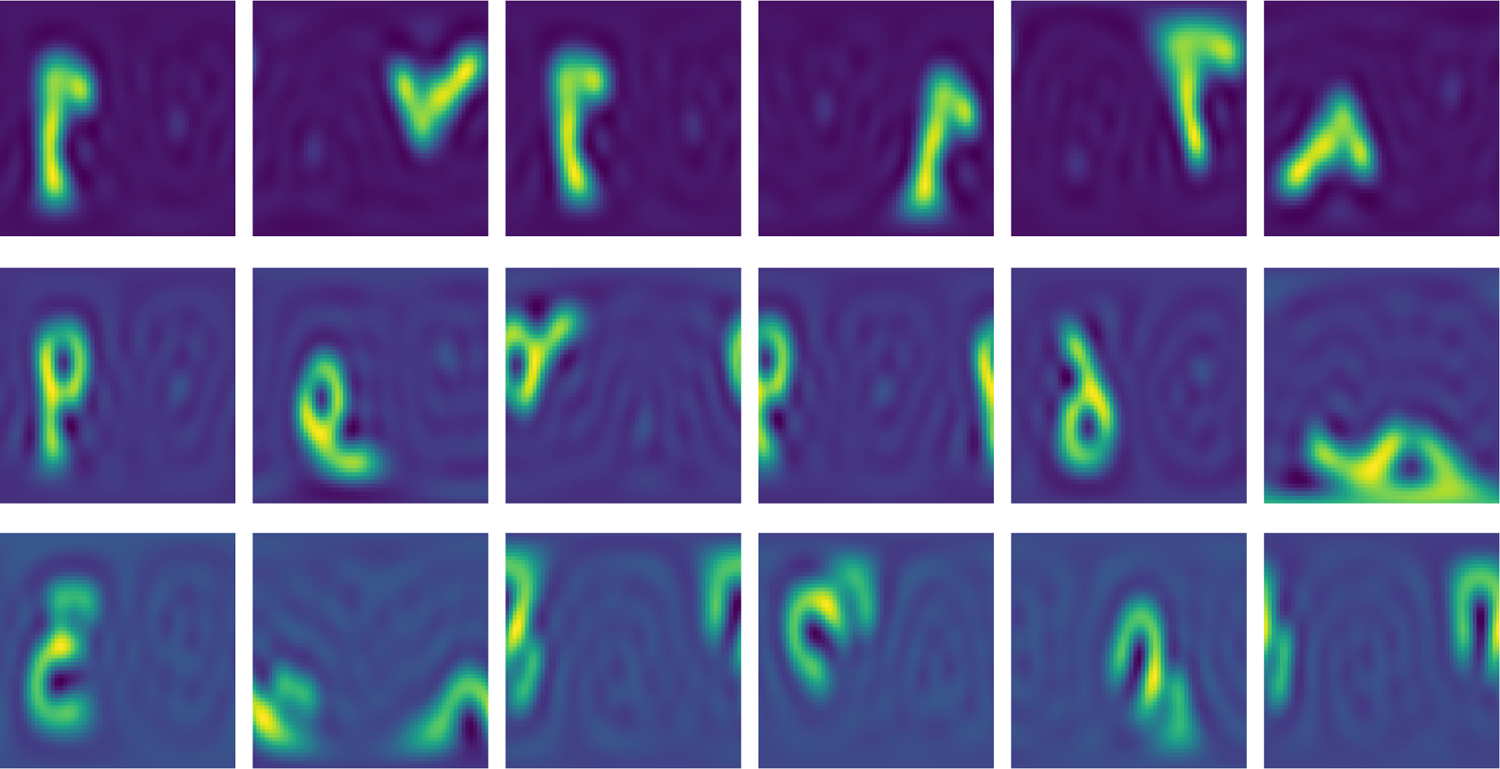
Visual proof of the disentanglement in the latent space of MNIST-on-the-sphere. For each row, the invariant embedding z is held fixed, and a different frame (i.e., the rotation matrix) is used. Frames are sampled randomly and differ across rows, with the exception of the first column, which is always the identity frame. Then, z and the frame are fed to the decoder and the Inverse Fourier Transform is used to generate the reconstructed spherical image, which is projected onto a plane for the ease of visualization. Modulo the distortions given by projecting the image onto a plane, it is clear that the invariant embedding contains all semantic information, and the frame solely determines the orientation of the image.

Interpretability of the latent space is also an important feature of a model. In Fig. 3 we provide empirical evidence of the disentanglement of the latent space into a rotation-invariant component and a rotation matrix. All eight models produce an invariant latent space that naturally clusters by digit identity, shown qualitatively for four of the models in Figs. 2(b) and Fig. S2 within the SM [28]. Any two sets of digits whose clusters are neighboring in the latent space [Fig. 2(b)] are digits that can be more easily confused with each other. The possibility of confusion is further exacerbated when considering that the latent embeddings used to describe the digits are *rotationally invariant*. Indeed, a handwritten 6 and a handwritten 9 are likely to look very similar to each other *up to an arbitrary rotation of each*. 4’s and 9’s too can be easily confused depending on how they are written. 0’s, on the other hand, are very different from any of the other digits, and so are 1’s, no matter how they are rotated. A qualitatively consistent pattern for separation of digit clusters is observed in the latent space of the Rot-Inv AE [33].

We measure the extent to which the invariant latent space forms clusters based on digit identity by applying K-means clustering (with 10 centroids) to the embeddings; Table I shows the standard metrics of purity [34] and V-measure [35] for these clusters. All trained models achieve much better clustering metrics compared to Rot-Inv AE [33], with the VAE models consistently outperforming the AE models. Crucially, the built-in SO(3) equivariance enables models trained on the nonrotated images to seamlessly generalize to images that have been randomly rotated, as seen by the equivalent performance between models trained and evaluated on the NR/R and R/R splits in Table I.

We also train a linear classifier (LC) to predict digit identity from invariant latent space descriptors, achieving a better accuracy compared to Rot-Inv AE with the same latent space size. We further observe marginal improvements of VAE over AE models in terms of classification accuracy. Using a K-nearest neighbor (KNN) classifier instead of LC further improves performance on the z=16 models (Table S3 within the SM [28]).

As H-VAE is a generative model, we generate random spherical images by sampling invariant latent embeddings from the prior distribution, and observing diversity in digit type and style [Fig. 2(c) and Figs. S7 and S8 within the SM [28]). We further assess the quality of the invariant latent space by generating images via linear interpolation of the invariant embeddings associated with two test images. The interpolated images present spatially consistent transitions [Fig. 2(d) and Fig. S13 within the SM [28]), which is a sign of a semantically well-structured latent space.

**FIG. 4.**
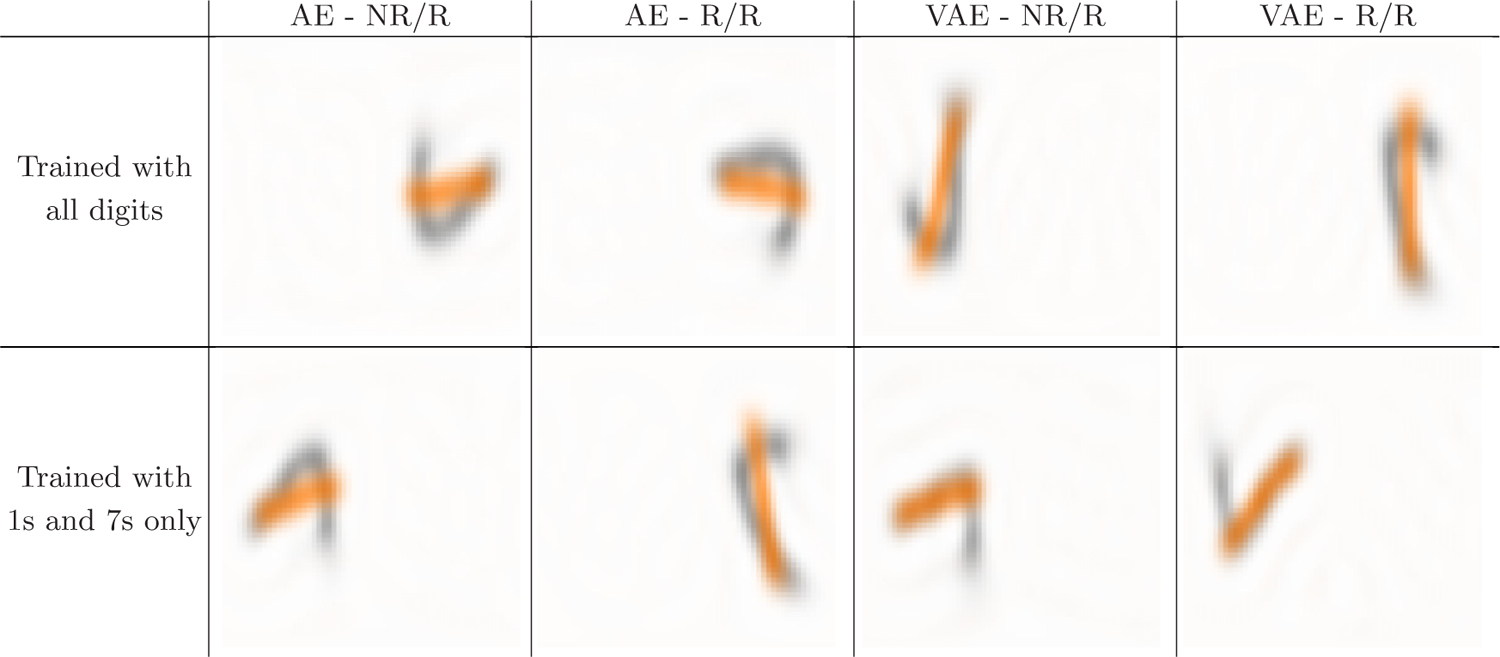
H-(V)AE implicitly learns to maximally overlap training images on MNIST-on-the-sphere. For each of the four models with z=16, we train a version using only images containing 1s and 7s. For each of the resulting eight models, we visualize the sum of training images of digits 1 and 7, when rotated to the canonical frame. We compute the sums of images with the same digit, and overlay them with different colors for ease of visualization. We test the hypothesis as whether H-(V)AE learns frames that align the training images such that they maximally overlap; we do so in two ways. First, if the hypothesis were true, all canonical images of the same digit should maximally or near-maximally overlap—since they have very similar shape—and thus, their overlays would look like a “smooth” version of that digit. Indeed, we find this statement to be true for all models irrespective of their training strategy. Second, we consider the alignment of images of different digits. We take 1s and 7s as examples given their similarity in shape. If the hypothesis were true, models trained with only 1s and 7s should align canonical 1s along the long side of canonical 7s; indeed we find this to be the case for the variational models, for which the embeddings are believed to be more semantically meaningful and are more robust to noise. The same alignment between 1s and 7s, however, does not necessarily hold for models trained with all digits. This is because maximizing overlap across a set of diverse shapes does not necessarily maximize the overlap within any independent pair of such shapes. Indeed, we find that canonical 1s and canonical 7s do not overlap optimally with each other for models trained with all digits. We note that these tests do not provide a formal proof, but rather empirical evidence of the characteristics of frames learned by H-(V)AE on the MNIST-on-the-sphere task.

To understand the meaning of the learned frames, we ask ourselves what the output of the decoder looks like if the frame is held constant; for simplicity, we set it equal to the 3 × 3 identity matrix. We find that the reconstructed elements tend to be aligned with each other and hypothesize that the model is implicitly learning to maximize the overlap between training elements, providing empirical evidence in Fig. 4. We call this frame the “canonical” frame. We note that it is possible to rotate original elements to the canonical frame thanks to the equivalence between the frame we learn and the rotation matrix within our implementation; in fact in our experiments, when visualizing reconstructed or sampled MNIST images, we first rotate them to the canonical frame for ease of visualization.

### B. Shrec17

The Shrec17 dataset consists of 51k colorless 3D models belonging to 55 object classes, with a 70/10/20 train/valid/test split [38]. We use the variant of the dataset in which each object is randomly rotated. Converting 3D shapes into spherical images preserves topological surface information, while significantly simplifying the representation. We follow Ref. [4] and project surface information for each image onto an enclosing spherical grid via a ray-casting scheme and apply ZFT [Eq. (2)] on these transformed images.

We train an AE and a VAE model on ZFT transformed data (Sec. A5c); Fig. S5 within the SM [28] shows the resulting latent embeddings for both H-AE and H-VAE on this dataset. Similarly to the MNIST dataset, we compute cosine loss, clustering metrics, and classification accuracy via a linear classifier. We also compute the standard Shrec17 object retrieval metrics via the latent space linear classifier’s predictions (see [38] for a description of the retrieval metrics). H-AE achieves the best classification and retrieval results for autoencoder-based models, and is competitive with supervised models despite the lower grid bandwidth and the small latent space (Table I). Using KNN classification instead of a linear classifier further improves performance (Table S4 within the SM [28]). H-VAE achieves slightly worse classification results but better clustering metrics compared to H-AE While. reconstruction loss is low, there is still significant margin of improvement.

### C. Structural embeddings of amino acid neighborhoods to predict function

Here, we provide a strong use case for H-(V)AE in structural biology. Specifically, we learn expressive embeddings for amino acids neighborhoods within protein structures that can be used to learn protein function.

#### Embeddings of amino acid conformations.

As a first, propedeutic step, we train H-(V)AE to reconstruct the 3D structure of individual amino acids, represented as atomic point clouds, extracted from protein structures in the Protein Data Bank (PDB) [39]. Residues of the same type have different conformations and naturally have noisy coordinates, making this problem a natural benchmark for our rotationally equivariant method.

We represent an amino acid by atom-type-specific clouds (C, O, N and S; we exclude H) centered at the residue’s Cα and compute the ZFT [Eq. (2)] with L=4 and N=20 within a radius of 10 Å from the residue’s Cα, and concatenate features of the same degree ℓ, resulting in a tensor with 940 coefficients. We train several H-AE and H-VAE with different architectures, but all with the latent space sizes z=2; see Sec. A5d for details.

**FIG. 5.**
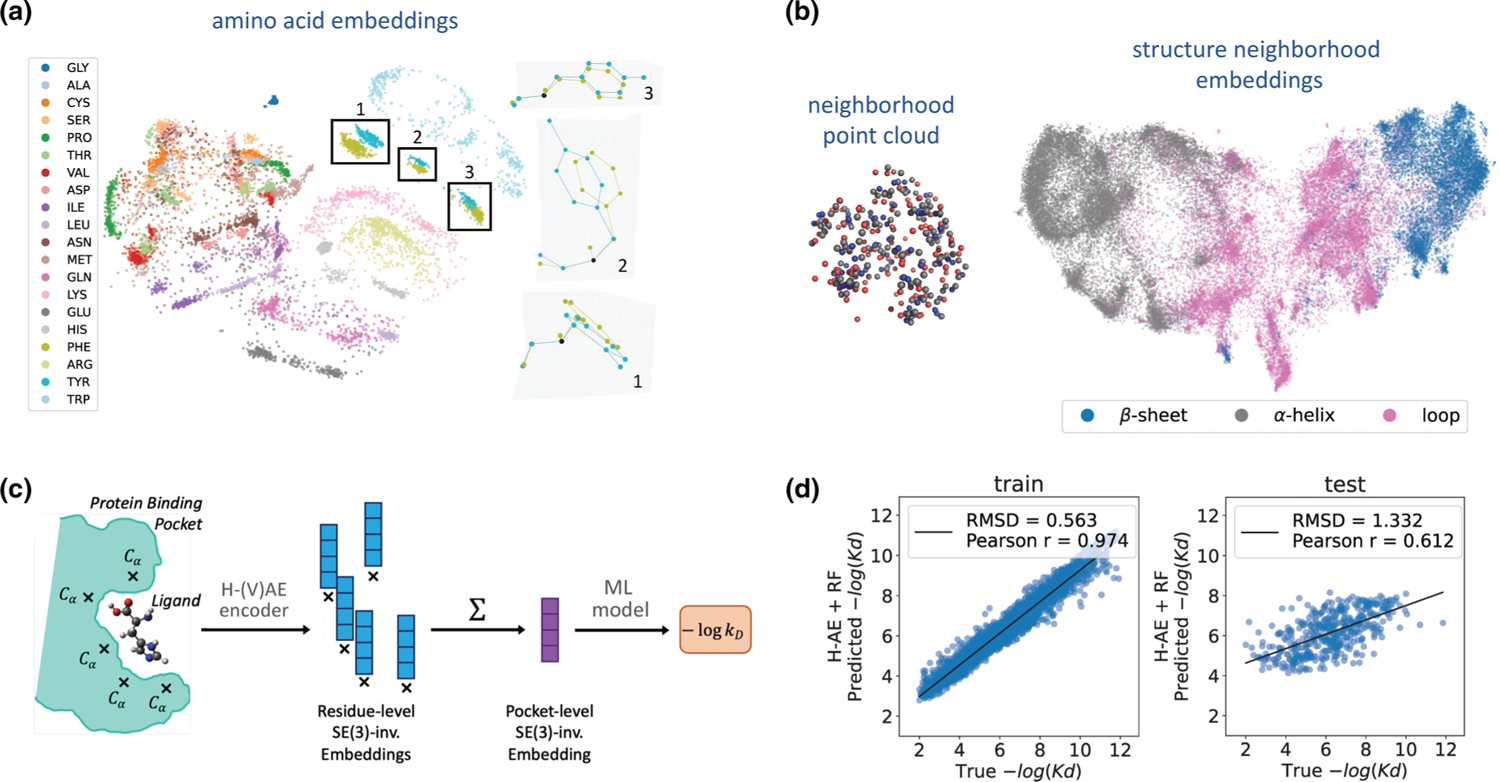
Structural embeddings to predict protein-ligand binding affinities with H-(V)AE. (a) H-VAE was trained to reconstruct the Fourier representation of 3D atomic point clouds representing amino acids (colors). The invariant latent space clusters by amino acid conformations. The highlighted clusters for PHE and TYR contain residue pairs with similar conformations; TYR and PHE differ by one oxygen at the end of their benzene rings. We compare conformations by plotting each residue in the standard backbone frame (right); x and y axes are set by the orthonormalized Cα-N and Cα-C vectors, and z axis is their cross product. For this plot, 1000 amino acids were used as training data, with network parameters: β=0.025 and z=2. (b) (Left) An example protein neighborhood (point cloud of atoms) of 10 Å around a central residue, used to train the H-AE models, is shown. (Right) 2D UMAP visualization of the 128-dimensional invariant latent space learned by H-AE trained on the protein structure neighborhoods with L=6 can separate neighborhoods by the secondary structure of their focal amino acid (colors). A linear classifier trained on 300 000 latent embeddings predicts secondary structure of the focal amino acid with 90% accuracy; see Figs. S18 and S19 within the SM [28] for a more detailed analysis of this latent space. Each point represents a neighborhood; see Sec. A5e for details on the network architecture and training procedure. (c) We use H-AE to extract the residue-level SO(3)-invariant embeddings in the binding pocket of a protein-ligand structure complex (data from PDBbind [36]). We then sum over these embeddings to form an SE(3)-invariant pocket embedding that is used as an input to a standard machine learning model to predict the binding affinity between the protein and the ligand. (d) The predictions on the protein-ligand binding affinities from (c) is shown against the true values for the training (left) and the test (right) sets. We use the data split provided by ATOM3D [37], which devises training and test sets respectively containing 3507, and 490 protein-ligand complexes, with maximum 30% sequence similarly between training and test proteins; see Table II for a comparison against state-of-the-art methods.

We consistently find that the latent space clusters by amino acid conformations [Fig. 5(a)], with sharper cluster separations as more training data is added (Figs. S15 and S16 within the SM [28]). We find that test reconstruction loss decreases with more training data but the reconstruction is accurate even with little training data (from 0.153 cosine loss with 400 training residues to 0.034 with 20 000); see Table S5 within the SM [28]. A similar trend is observed for KNN-based classification accuracy of residues (from 0.842 with 400 training residues to 0.972 with 20 000); (Table S5 within the SM [28]). Notably, an untrained model, while achieving random reconstruction loss, still produces an informative invariant latent space (0.629 residue type accuracy), suggesting that the forced SO(3) invariance grants a “warm start” to the encoder. We do not find significant improvements in latent space classification by training with a variational objective, and present ablation results in Table S6 within the SM [28].

#### Embeddings of amino acid structure neighborhoods.

The structural neighborhood surrounding an amino acid provides a context for its function (e.g., whether it takes part in interaction with other proteins at a protein-protein interface or not). Indeed, our previous study has shown that supervised learning algorithms can accurately classify focal amino acids based on the composition of their surrounding neighborhoods [40]. Here, we train H-(V)AE to reconstruct residue-specific protein *neighborhoods*—which we define as the point clouds of atoms within 10 Å of a residue’s Cα—across the protein universe [Fig. 5(a)]. We extract these protein neighborhoods from all proteins in ProteinNet [41]. We then construct each neighborhood’s Fourier representation by computing the ZFT [Eq. (2)] over the point clouds associated with each atom type within the neighborhood (C, N, O, and S) and concatenating atom-specific features of the same degree ℓ.

We train several H-AE models with varying architectures with different maximum spherical degree L and latent space sizes z (see details in Sec. A5e); note that we do not experiment with variational models for this task. H-AE shows strong reconstruction ability, but its accuracy worsens with smaller latent space sizes and higher maximum spherical degree L (Table S7 within the SM [28]). Notably, the learned latent space is smoothly structured according to the geometric features of the neighborhoods, such as the presence of different secondary structure components [Fig. 5(b), and Fig. S18 within the SM [28]) and the number of atoms comprising a neighborhood (Fig. S19 within the SM [28]).

#### Predicting protein-ligand binding affinity (LBA).

The binding interaction between proteins and ligands should be primarily determined by the composition of the protein’s binding pocket in complex with the ligand. Therefore, we hypothesized that the inferred protein structure embeddings [Fig. 5(b)] should contain information about protein-ligand binding interactions, if the neighborhood is defined along the binding pocket and contains atoms from the ligand. To test this hypothesis, we follow the pipeline in Fig. 5(c). Specifically, given a protein-ligand structure complex, we identify residues in the binding pocket (i.e., residues with C-α within 10 Å of the ligand) and extract their structure neighborhoods, which include atoms from both the protein and the ligand. We then pass the residue-centered neighborhoods of the binding pocket through a trained H-AE’s encoder to extract their rotationally invariant embeddings. We highlight that, since the neighborhood centers are well defined at the residues’ $C\alpha^{\prime }s$, the embeddings are not only rotationally invariant about their center, but also translationally invariant with the respect to translations of the whole system, i.e., they are effectively SE(3) invariant.

Since protein-ligand binding affinity is an extensive quantity in the number of interacting residues, we construct a pocket embedding by summing over residue-level embeddings; the resulting pocket embedding is SE(3) invariant, reflecting the natural symmetry of the LBA task. We use these pocket embeddings as feature vectors to train simple machine learning models to predict protein-ligand binding affinities.

To test the performance of our method, we use the LBA dataset in ATOM3D [37] that provides the PDB structure of the protein-ligand complex together with either the measured dissociation constant $K_{d}$ or the inhibition constant $K_{i}$; see Sec. A6 for further details. To map between the learned pocket embeddings to the log dissociation (or inhibition) constants we train both a simple linear and a random forest regressor on a training sets provided by ATOM3D. Figure 5(d) shows the performance of the model with the random forest regressor and Table II provide a detailed benchmark of our methods against prior approaches. The linear model achieves competitive results, whereas the random forest regressor achieves state-of-the-art.

These results demonstrate the utility of unsupervised learning for residue-level protein structure representations in predicting complex protein functions. Most of the competing structure-based methods for LBA (Table II) learn complex graph-based functions on top of simple atomic representation, whereas our method uses simpler machine learning models over rich residue-level representations. Notably, ProtMD (the method competitive to ours) performs a pretraining scheme using expensive molecular dynamics simulations that informs the model about conformational flexibility, information that our method does not have access to. Given the computational cost of training complex atom-level graph-based models, our residue-based approach can offer a more viable alternative for modeling large protein interfaces.

## V. DISCUSSION

In this paper, we have developed the first end-to-end SO(3)-equivariant unsupervised algorithm, termed “holographic (variational) autoencoder” [H-(V)AE], suitable for data distributed in three dimensions around a given central point. The model learns an invariant embedding describing the data in a “canonical” orientation alongside an equivariant frame describing the data’s original orientation relative to the canonical one.

Prior studies have attempted to learn representations that are invariant to certain transformations. For example, in Refs. [42,43] general “shape” embeddings are learned by characterizing a separate “deformation” embedding. However, these networks are not explicitly equivariant to the transformations of interest. Others proposed to learn an exactly invariant embedding alongside an approximate (but not equivariant) group action to align the input and the reconstructed data. For example, Mehr *et al*. [44] learns in quotient space by pooling together the latent encodings of copies of the data that have been transformed with sampled group actions, and back-propagate the minimum reconstruction loss between the decoded element and the transformed copies of the data. This approach is best suited for discrete and finite groups, for which it does not require approximations, and it is computationally expensive as it is akin to data augmentation. Lohit *et al*. [33] construct an SO(3)-invariant autoencoder for spherical signals by learning an invariant latent space and minimizing a loss, which first finds the rotation that best aligns the true and reconstructed signals. Although this approach is effective for nondiscrete data, it still manually imposes rotational invariance, and can only reconstruct signals up to a global rotation. In contrast, H-(V)AE is fully equivariant and only requires simple MSE for reconstruction of data in its original orientation.

A small body of work went beyond invariance to develop equivariant autoencoders. Several methods construct data and group-specific architectures to autoencode data equivariantly, learning an equivariant representation in the process [45,46]. Others use supervision to extract class-invariant and class-equivariant representations [47]. A recent theoretical paper proposes to train an encoder that encodes elements into an invariant embedding and an equivariant group action, then using a standard decoder that uses the invariants to reconstruct the elements in a canonical form, and finally applying the learned group action to recover the data’s original form [15]. Our method in SO(3) is closely related to this paper, with the crucial differences that our network is end-to-end rotationally equivariant in that we use an equivariant decoder, and that we learn to reconstruct the Fourier encoding of the data. A more detailed comparison of the two approaches and the benefits of our fully equivariant approach can be found in the Appendix A3 and in Table III.

Recently, generative models for 3D atomic point clouds that are equivariant to Euclidean transformation have been developed for molecules [11,16] and protein [17,18], using normalizing flows [11], and diffusion processes [16–18]. Our paper is partly related to these, but with some crucial differences. First, our method is not limited to only point clouds, but is designed for general 3D objects with a specified center, including spherical images. Second, the use of an autoencoder architecture makes our method suitable for learning compressed and low-dimensional representation of complex data. Importantly, this reduced representations can be used in semisupervised learning tasks when annotated data is scarce, such the task to predict protein-ligand binding affinity. By contrast, the latent space of normalizing flows and diffusion-based models must have the same size as the data, making them unsuitable for semisupervised learning purposes.

There is also a diverse body of literature on using Fourier transforms and and CG tensor products to construct representations of atomic systems that are invariant/equivariant to euclidean symmetries [48–50], but without reducing the dimensionality of the representations in a data-driven way.

H-(V)AE’s learned embeddings are highly expressive. For example, we used the learned invariants to achieve state-of-the-art unsupervised clustering and classification results on various spherical image datasets. By making our model variational in its invariant latent space, we enhanced the quality of clustering and made the model generative. Our model is defined fully in spherical Fourier space, and thus, can reach a desired expressiveness without a need for excessive computational resources.

H-(V)AE also produces rich residue-level representations of local neighborhoods in protein structures, which we use as embeddings for downstream structure-based tasks such as ligand binding affinity prediction. Indeed, H-(V)AE representations paired with a simple random forest regressor achieve state-of-the-art results on learning the binding affinity between proteins and small molecule ligands.

More broadly, we expect that H-(V)AE can be used to extract rich, symmetry-aware features from local neighborhoods in spherical images and complex 3D objects, to be used in more complex downstream tasks that benefit from the symmetry constraints. For example, we expect our method can be leveraged for modeling diffusion MRI data, for which rotation-equivariant methods have recently proven to be highly beneficial [51]. In structural biology, we expect our method to be useful for coarse-graining full-atom representations of protein structures—or other biomolecules—to facilitate structure-based predictions of function. For example, a large protein graph can be coarse grained by substituting its full-atom representation with rich embeddings of local structural neighborhoods learned from an unsupervised model. With an added supervised step, these coarse-grained embeddings can be leveraged to predict complex protein functions, as we show for predicting ligand binding affinity. This approach is akin to using protein embeddings for sequence data, learned by language models, to inform (few-shot) predictions for protein function [52].

Currently H-(V)AE is limited by its ability to reconstruct features associated with higher spherical degrees (types) ℓ (Fig. S17 within the SM [28]), indicating loss of information for the fine-grained features of the data. This may be due to the fact that in our current architecture features associated with all types are simultaneously processed to form the set of invariants (ℓ=0) in the latent space. We hypothesize that information originating from lower ℓ types could be more easily processed into the ℓ=0 features, and thus, they may be more preferentially represented in the invariants of the latent space. This information integration imbalance could be remedied by developing a multiscale autoencoder, similar to Refs. [20,21], which separately encodes and processes the features associated with different types. We leave the pursuit of this idea to future work.

## Supplementary Material

SI

## Figures and Tables

**TABLE I.** Performance metrics on MNIST-on-the-sphere and Shrec17. Reconstruction Cosine loss, clustering metrics (purity and V-measure), classification accuracy in the latent space using a linear classifier, and retrieval metrics (Shrec17 only) are shown. For MNIST, we follow Ref. [4] to create the MNIST-on-the-sphere dataset by projecting data from the planar MNIST on a discrete unit sphere, using the Driscoll-Healey method with a bandwidth (bw) of 30. We then map the images to steerable tensors via the Zernike Fourier transform (ZFT) with L=10, and a constant radial function $R_{\ell }^{n}=1$, resulting in tensors with 121 coefficients. We train eight models with different sizes of latent spaces z (16 vs 120) and model types (AE vs VAE). For Shrec17, we follow Ref. [4] and project surface information from each model onto an enclosing Driscoll-Healey spherical grid with a bandwidth of 90 via a ray-casting scheme, generating spherical images with 6 channels. We then apply the ZFT with L=14 and a constant radial function $R_{\ell }^{n}=1$ to each channel individually, resulting in a tensor with 1350 coefficients. We only report scores presented in the corresponding papers, and only the best-performing supervised method from the literature; see Fig. S14 within the SM [28] for visualization of the latent embeddings for the Shrec17 dataset.

Dataset	Type	Method	z	bw	Cosine	Purity	V-meas.	Class. Acc.	P@N	R@N	F1@N	mAP	NDCG
	Supervised	[30] NR/R		30				0.993					
		[33] NR/R	120	30		0.40	0.35	**0.894**					
		H-AE NR/R (Ours)	120	30	0.017	0.59	0.51	0.920					
		H-AE R/R (Ours)	120	30	0.015	0.65	0.52	0.916					
MNIST	Unsupervised	H-AE NR/R (Ours)	16	30	0.031	0.66	0.55	0.850					
		H-AE R/R (Ours)	16	30	0.030	0.61	0.52	0.844					
		H-VAE NR/R (Ours)	120	30	0.039	0.73	0.61	**0.923**					
		H-VAE R/R (Ours)	120	30	0.041	0.70	0.60	**0.923**					
		H-VAE NR/R (Ours)	16	30	0.068	0.73	0.60	0.878					
		H-VAE R/R (Ours)	16	30	0.067	0.69	0.57	0.855					
Shrec17	Supervised	[7]		128					0.717	0.737		0.685	
		[30]		128					0.719	0.710	0.708	0.679	0.758
	Unsupervised	[33]	120	30		0.41	0.34	0.578	0.351	0.361	0.335	0.215	0.345
		H-AE (Ours)	40	90	0.130	0.50	0.41	**0.654**	**0.548**	**0.569**	**0.545**	**0.500**	**0.597**
		H-VAE (Ours)	40	90	0.151	**0.52**	**0.42**	0.631	0.512	0.537	0.512	0.463	0.568

**TABLE II.** Results on the Ligand Binding Affinity task. Prediction accuracies using H-AE embeddings with linear regression (L.R.), and random forest (R.F.) regression are benchmarked against other methods. We choose the H-AE model with best RMSD on validation split, which is the model with L=6 and z=128 for both linear regression and random forest. For each set of predictions, we use an ensemble of ten regressors as we noted a small but consistent improvement in performance. Best scores are in **bold** and second-best scores are underlined. H-AE+R.F. delivers state-of-the-art predictions. Methods are ordered by date of release; see Table IV for a more extended comparison.

	Ligand Binding Affinity 30% Similarity		
Model	RMSD ↓	Pearson’s r ↑	Spearman’s r ↑
DeepDTA	1.565	0.573	0.574
3DCNN	1.414 ± 0.021	0.550	0.553
GNN	1.570 ± 0.025	0.545	0.533
MaSIF	1.484 ± 0.018	0.467 ± 0.020	0.455 ± 0.014
EGNN	1.492 ± 0.012	0.489 ± 0.017	0.472 ± 0.008
GBPNet	1.405 ± 0.009	0.561	0.557
EGNN + PLM	1.403 ± 0.013	0.565 ± 0.016	0.544 ± 0.005
ProtMD	1.367 ± 0.014	0.601 ± 0.036	0.587 ± 0.042
H-AE + L.R.	1.397 ± 0.019	0.560 ± 0.017	0.568 ± 0.018
H-AE + R.F.	**1.332** ± **0.012**	**0.612** ± **0.009**	**0.619** ± **0.009**

**TABLE III.** Performance comparison between our H-(V)AE and a H-(V)AE with Ref. [15]’s nonequivariant decoder formulation, on the MNIST-on-the-sphere dataset. The nonequivariant decoders are constructed as simple MLPs with SiLU nonlinearities, with the following hidden layer sizes: [32, 64, 128, 160, 256]. We keep the number of parameters approximately the same to make model comparison fair, but we do not tune the architecture of the invariant decoders. All other training details are kept the same (Sec. A5).

Method	z	Speed	MSE	Cosine	Purity	V-meas.	LC Class. Acc.	KNN Class. Acc.
H-AE NR/R [ours]	16	**1.0**	1.2 × 10^−3^	0.031	**0.66**	**0.55**	**0.850**	0.902
H-AE unconst. decoder NR/R	16	1.3	**1.1** × **10**^−**3**^	**0.028**	0.62	0.53	0.838	**0.907**
H-VAE NR/R [ours]	16	**1.0**	**2.8** × **10**^−**3**^	**0.068**	**0.73**	**0.60**	**0.878**	**0.897**
H-VAE unconst. decoder NR/R	16	1.3	2.9 × 10^−3^	0.073	0.68	0.56	0.850	0.872

**TABLE IV.** Comprehensive benchmarking results on the Ligand Binding Affinity task with Atom3D’s 30% similarity split. Models are sorted by date of release. In addition to the H-AE informed models, we also report the performance of baseline models that only use the SO(3)-invariant (ℓ=0) component of each neighborhood’s Zernike transform (Zernike Inv.). H-AE consistently outperforms this baseline, indicating that the SO(3)-invariant information from the higher-degree features extracted by H-AE are informative for this regression task. Best scores are in **bold** and second-best scores are underlined. Errors for our models are computed as the standard deviation in prediction by 10 machine learning models trained with bootstrapped data.

	Ligand Binding Affinity 30% Similarity			
Method	RMSD ↓	Pearson’s r ↑	Spearman’s r ↑	Kendall’s *τ* ↑
DeepDTA [59]	1.565	0.573	0.574	
DeepAffinity [60]	1.893 ± 0.650	0.415	0.426	
Cormorant [61]	1.568 ± 0.012	0.389	0.408	
ProtTrans [62]	1.544 ± 0.015	0.438 ± 0.053	0.434 ± 0.058	
3DCNN [37]	1.414 ± 0.021	0.550	0.553	
GNN [37]	1.570 ± 0.025	0.545	0.533	
MaSIF [63]	1.484 ± 0.018	0.467 ± 0.020	0.455 ± 0.014	
DGAT [64]	1.719 ± 0.047	0.464	0.472	
DGIN [64]	1.765 ± 0.076	0.426	0.432	
DGAT-GCN [64]	1.550 ± 0.017	0.498	0.496	
GVP-GNN [65]	1.648 ± 0.014	0.213 ± 0.013	0.164 ± 0.009	0.110 ± 0.012
EGNN [11]	1.492 ± 0.012	0.489 ± 0.017	0.472 ± 0.008	0.329 ± 0.014
HoloProt [66]	1.464 ± 0.006	0.509 ± 0.002	0.500 ± 0.005	
GBPNet [67]	1.405 ± 0.009	0.561	0.557	
EGNN + PLM [68]	1.403 ± 0.013	0.565 ± 0.016	0.544 ± 0.005	0.379 ± 0.007
ProtMD [69]	1.367 ± 0.014	0.601 ± 0.036	0.587 ± 0.042	
Zernike Inv. + linear regression	1.455 ± 0.005	0.513 ± 0.005	0.516 ± 0.006	0.357 ± 0.005
Zernike Inv. + random forest	1.361 ± 0.011	0.587 ± 0.009	0.584 ± 0.010	0.408 ± 0.008
H-AE + linear regression	1.397 ± 0.019	0.560 ± 0.017	0.568 ± 0.018	0.397 ± 0.016
H-AE + random forest	**1.332** ± **0.012**	**0.612** ± **0.009**	**0.619** ± **0.009**	**0.436** ± **0.006**

## APPENDIX

### 1. Expanded background on SO(3) equivariance

#### a. Invariance and Equivariance

Let f:X→Y be a function between two vector spaces and G a group, where G acts on X and via representation $D_{X}$ and on Y via representation $D_{Y}$. Then, f is said to be G-equivariant iff $f\left(D_{X}(g)x\right)=D_{Y}(g)f(x),\forall x\in X\wedge \forall g\in G$. We note that invariance is a special case of equivariance where $D_{Y}(g)=I,\forall g\in G$.

#### b. Group representations and the irreps of SO(3)

Groups can concretely act on distinct vector spaces via distinct group representations. Formally, a group representation defines a set of invertible matrices $D_{X}(g)$ parameterized by group elements g∈G, which act on vector space X. As an example, two vector spaces that transform differently under the 3D rotation group SO(3)—and thus have different group representations—are scalars, which do not change under the action of SO(3), and 3D vectors, which rotate according to the familiar 3D rotation matrices.

A special kind of representation for any group are the irreducible representations (irreps), which are provably the “smallest” nontrivial (i.e., they have no nontrivial group-invariant subspaces) representations. The irreps of a group are special because it can be proven that any finite-dimensional unitary group representation can be decomposed into a direct sum of irreps [24]. This applies to SO(3) as well, whose irreps are the Wigner D-matrices, which are (2ℓ+1×2ℓ+1)-dimensional matrices, each acting on a (2ℓ+1)-dimensional vector space,

(A1)
$$D_{\ell }\left(g\right)\;\text{for}\;\ell =0,1,2,\ldots$$

Therefore, every element of the SO(3) group acting on any vector space can be represented as a direct sum of Wigner D-matrices.

#### c. Steerable features

A G-steerable vector is a vector x∈X that under some transformation group G, transforms via matrix-vector multiplication $D_{X}(g)x$; here, $D_{X}(g)$ is the group representation of g∈G. For example, a vector in 3D Euclidean space is SO(3) steerable since it rotates via matrix-vector multiplication using a rotation matrix.

However, we can generalize 3D rotations to arbitrary vector spaces by employing the irreps of SO(3). We start by defining a degree-ℓ feature as a vector that is SO(3) steerable by the ℓth Wigner D-matrix $D_{\ell }$. Given the properties of irreps, we can represent any SO(3)-steerable vector as the direct sum of two or more independent degree-ℓ features, e.g., $x=x_{\ell_{1}}⊕x_{\ell_{2}}⊕\ldots ⊕x_{\ell_{n}}$. The resulting vector, which we refer to as a *tensor* to indicate that it is composed of multiple individually steerable vectors, is SO(3) steerable via the direct sum of Wigner D-matrices of corresponding degrees. This tensor is a block-diagonal matrix with the Wigner D-matrices along the diagonal, $D(g)=D_{\ell_{1}}(g)⊕D_{\ell_{2}}(g)⊕\ldots ⊕D_{\ell_{n}}(g)$.

#### d. Spherical harmonics and the Spherical Fourier Transform

Spherical harmonics are a class of functions that form a complete and orthonormal basis for functions f(θ,ϕ) defined on a unit sphere; θ and ϕ are the azimuthal and the polar angles in the spherical coordinate system. In their complex form, spherical harmonics are defined as

(A2)
$$Y_{\ell m}\left(\theta ,\phi \right)=\sqrt{\frac{2n+1}{4\pi }\frac{\left(n-m\right)!}{\left(n+m\right)!}}e^{im\phi }P_{\ell }^{m}\left(\text{cos}\;\theta \right)$$

where ℓ is a non-negative integer (0⩽ℓ) and m is an integer within the interval $-\ell ⩽m⩽\ell .P_{\ell }^{m}(cos\theta )$ is the Legendre polynomial of degree ℓ and order m, which together with the complex exponential $e^{im\phi }$ define sinusoidal functions over the angles θ and ϕ in the spherical coordinate system. Spherical harmonics are used to describe angular momentum in quantum mechanics.

Notably, spherical harmonics also form a basis for the irreps of SO(3), i.e., the Wigner D-matrices. Specifically, the SO(3) group acts on the ℓth spherical harmonic via the ℓth Wigner D-matrix,

(A3)
$$Y_{\ell m}\left(\theta ,\phi \right)\overset{g\in SO\left(3\right)}{\to }\overset{\ell }{\underset{m^{\prime }=-\ell }{\sum }}D_{\ell }^{m^{\prime }m}\left(g\right)Y_{\ell m^{\prime }}\left(\theta ,\phi \right).$$

Therefore, any data encoded in a spherical harmonics basis is acted upon by the SO(3) group via a direct sum of the Wigner D-matrices corresponding to the basis functions being used. Using our nomenclature, any such data encoding constitutes a steerable tensor. We can thus map any function f(θ,ϕ) defined on a sphere into a steerable tensor using the spherical Fourier transform (SFT),

(A4)
$${\hat{f}}_{\ell m}=\int_{0}^{2\pi }\int_{0}^{\pi }f\left(\theta ,\phi \right)Y_{\ell m}\left(\theta ,\phi \right)\;\text{sin}\;\theta \;d\theta \;d\phi .$$

The signal can be reconstructed in the real space using the corresponding inverse Fourier transform. For computational purposes, we truncate Fourier expansions at a maximum angular frequency L, which results in an approximate reconstruction of the signal $\tilde{f}(\theta ,\varphi )$ up to the angular resolution allowed by L,

(A5)
$$\tilde{f}\left(\theta ,\varphi \right)=\overset{L}{\underset{\ell =0}{\sum }}\overset{\ell }{\underset{m=-\ell }{\sum }}{\hat{f}}_{\ell m}Y_{\ell m}\left(\theta ,\phi \right).$$

Here, ${\overset{ˆ}{f}}_{\ell m}$ are the functions’ spherical Fourier coefficients.

#### e. Zernike polynomials and the Zernike Fourier transform

To encode a function ρ(r,θ,ϕ) with both radial and angular components, we use Zernike Fourier transform,

(A6)
$${\hat{Z}}_{\ell m}^{n}=\int \rho \left(r,\theta ,\phi \right)Y_{\ell m}\left(\theta ,\phi \right)R_{\ell }^{n}\left(r\right)dV$$

where $R_{\ell }^{n}(r)$ is the radial Zernike polynomial in 3D defined as

(A7)
Rℒn(r)=(−1)n−ℒ22n+3(n+ℒ+32−1n−ℒ2)|r|ℒ×F21(−n−12,n+ℒ+32;ℒ+32;|r|2).

Here, F21(⋅) is an ordinary hypergeometric function, and n is a non-negative integer representing a radial frequency, controlling the radial resolution of the coefficients. $R_{\ell }^{n}(r)$ is nonzero only for even values of n-ℓ⩾0. Zernike polynomials form a complete orthonormal basis in 3D, and therefore, can be used within a Fourier transform to expand and retrieve any 3D shape, if large enough ℓ and n coefficient are used. We refer to the Fourier transform of Eq. (A6) as the Zernike Fourier transform (ZFT).

To represent point clouds, a common choice for the function ρ(r)≡ρ(r,θ,ϕ) is the sum of Dirac δ functions centered at each point,

(A8)
$$\rho \left(r\right)=\underset{i\in \text{points}}{\sum }\delta \left(\rho \left(r_{i}\right)-\rho \left(r\right)\right)$$

This choice is powerful because the forward transform has a closed-form solution that does not require a discretization of 3D space for numerical computation. Specifically, the ZFT of a point cloud follows

(A9)
$${\hat{Z}}_{\ell m}^{n}=\underset{i\in \text{points}}{\sum }R_{n}^{\ell }\left(r_{i}\right)Y_{\ell m}\left(\theta_{i},\varphi_{i}\right).$$

Similar to SFT, we can reconstruct the data using inverse ZFT and define approximations by truncating the angular and radial frequencies at L and N, respectively,

(A10)
$$\tilde{\rho }\left(r,\theta ,\varphi \right)=\overset{L}{\underset{\ell =0}{\sum }}\overset{\ell }{\underset{m=-\ell }{\sum }}\overset{N}{\underset{n}{\sum }}{\hat{Z}}_{\ell m}^{n}R_{\ell }^{n}\left(r\right)Y_{\ell m}\left(\theta ,\varphi \right).$$

The use of other radial bases is possible within our framework, as long as they are complete. Orthonormality is also desirable as it ensures that each basis encodes different information, resulting in a more efficient encoding of the coefficients. We use Zernike polynomials following Ref. [53], which demonstrates that encoding with Zernike polynomials result in a faster convergence compared to the radial basis functions localized at different radii, as well as most other orthogonal harmonic bases, with the exception of Logan-Shepp. “Faster convergence” indicates that fewer frequencies are required to encode the same information. Reference [50] also uses Zernike to construct invariant descriptors of atomic environments. However, this choice is not unique and other special functions such as Bessel functions can be used to encode the data [10].

### 2. Holographic-(V)AE (H-(V)AE) details

#### a. Linearity

Let us consider a feature $h_{\ell }$ containing C features of the same degree $\ell .h_{\ell }$ can be represented as a C×(2ℓ+1) matrix where each row constitutes an individual feature. Then, we learn weight matrix $W_{\ell }\in R^{C\times K}$ that linearly maps $h_{\ell }$ to ${\overset{‾}{h}}_{\ell }\in R^{K\times (2\ell +1)}$,

(A11)
$${\bar{h}}_{\ell }=W_{\ell }^{T}h_{\ell }.$$

#### b. Efficient Tensor Product (ETP)

##### Channel-wise tensor product nonlinearity.

We effectively compute C tensor products, each between features belonging to the same channel c, and concatenate all output features of the same degree. In other words, features belonging to different channels are not mixed in the nonlinearity; the across-channel mixing is instead done in the linear layer. This procedure reduces the computational time and the output size of the nonlinear layers with respect to the number of channels C, from $𝒪\left(C^{2}\right)$ for a “fully connected” tensor product down to 𝒪(C). The number of learnable parameters in a linear layer are proportional to the size of the output space in the preceding nonlinear layer. Therefore, reducing the size of the nonlinear output substantially reduces the complexity of the model and the number of model parameters. This procedure also forces the input tensor to have the same number of channels for all degrees. We refer the reader to Ref. [30] for further details and for a nice visualization of this procedure.

##### Minimum spanning tree (MST) subset for degree mixing.

To compute features of the same degree $\ell_{3}$ using the CG tensor product, pairs of features of varying degrees may be used, up to the rules of the CG tensor product. Specifically, pairs of features with any degree pair $\left(\ell_{1},\ell_{2}\right)$ may be used to produce a feature of degree $\ell_{3}$ as long as $\left|\ell_{1}-\ell_{2}\right|⩽\ell_{3}⩽\ell_{1}+\ell_{2}$. Features of the same degree are then concatenated to produce the final equivariant (steerable) output tensor.

Since each produced feature (often referred to as a “fragment” in the literature [6,30]) is independently equivariant, computing only a subset of them still results in an equivariant output, albeit with lower representational power. Reducing the number of computed fragments is desirable since their computation cannot be easily parallelized. In other words, to reduce complexity we should identify a small subset of fragments that can still offer sufficient representational power. In this paper we adopt the “MST subset” solution proposed in [30], which adopts the following strategy: when computing features of the same degree $\ell_{3}$, exclude the degree pair ($\ell_{0}\left.{,\ell }_{2}\right)$ if the $\left(\ell_{0},\ell_{1}\right)$ and the $\left(\ell_{1},\ell_{2}\right)$ pairs have already been computed. The underlying assumption behind this solution is that the last two pairs already contain some information about the first pair, thus making its computation redundant.

The resulting subset of pairs can be efficiently computed via the minimum spanning tree of the graph describing the possible pairs used to generate features of a single degree $\ell_{3}$, given the maximum desired degree $\ell_{\text{max}}$. As multiple such trees exist, we choose the one minimizing the computational complexity by weighting each edge (i.e., each pair) in the graph accordingly [edge $\left(\ell_{1},\ell_{2}\right)$ gets weight $\left(2\ell_{1}+1\right)\left(2\ell_{2}+\right.\;1)]$. The subset is also augmented to contain all the pairs with same degree to inject more nonlinearity. This procedure reduces the complexity in number of pairs with respect to $\ell_{\text{max}}$ from $𝒪\left(\ell_{max}^{2}\right)$—when all possible pairs are used—down to $𝒪\left(\ell_{max}\right)$. We refer the reader to Ref. [30] for more details.

#### c. Batch Norm

Let us consider a batch of steerable tensors h, which we index by batch b, degree ℓ, order m and channel c. During training, we compute a batch-averaged norm for each degree ℓ and each channel c as

(A12)
$$N_{\ell }^{c}=\frac{1}{B}\overset{B}{\underset{b=1}{\sum }}\frac{1}{2\ell +1}\overset{\ell }{\underset{m=-\ell }{\sum }}{\left({\hat{h}}_{\ell m}^{cb}\right)}^{2}.$$

Similar to standard batch normalization, we also keep a running estimate of the training norms $N_{\ell }^{c,tr(i)}$ using momentum ξ, set to 0.1 in all our experiments,

(A13)
$$N_{\ell }^{c,tr\left(i\right)}=\xi N_{\ell }^{c}+\left(1-\xi \right)N_{\ell }^{c,tr\left(i-1\right)}.$$

We then update the features of the steerable tensor using the real batch-averaged norms during training, and the running batch-averaged norms during testing, together with a learned affine transformation,

(A14)
$$\bar{{\hat{h}}_{\ell m}^{cb}}=\frac{{\hat{h}}_{\ell m}^{cb}}{\sqrt{N_{\ell }^{c}}}w_{\ell }^{c}\;\text{training},$$

(A15)
$$\bar{{\hat{h}}_{\ell m}^{cb}}=\frac{{\hat{h}}_{\ell m}^{cb}}{\sqrt{N_{\ell }^{c,tr\left(i\right)}}}w_{\ell }^{c}\;\text{evaluation}.$$

#### d. Signal Norm

Similar to for batch norm above, let us consider a batch of steerable tensors h, which we index by batch b, degree ℓ, order m, and channel c. Formally, the total norm for an individual tensor h is computed as

(A16)
$$N_{\text{tot}}=\underset{\ell }{\sum }\frac{\sum_{c}\sum_{m=-\ell }^{\ell }{\left({\hat{h}}_{\ell m}^{c}\right)}^{2}}{2\ell +1}.$$

Then, the tensor features are updated as $\bar{{\hat{h}}_{\ell m}^{c}}={\hat{h}}_{\ell m}^{c}w_{\ell }/\sqrt{N_{\text{tot}}}$, where $w_{\ell }$ is a degree-specific affine transformation for added flexibility

#### e. Network architecture

Certain features of our problem formulation pose constraints upon the specific design choice of the network. For example, within the decoder, the maximum degree $\ell_{max,b}$ that can be outputted by each block b is constrained by the sparsity of the CG tensor product. Specifically, $\ell_{max,b}⩽2^{b}$ where b ranges from 1 (first block) to B (last block). Since we need to reconstruct features up to degree L in the decoder, we arrive at a lower bound for the number of blocks in the decoder set by $\ell_{max,B}⩾L$, or $B⩾{log}_{2}L$. In our experiments, we set $\ell_{max,b}=min\left\{2^{b},L\right\}$ and do not let $\ell_{max,b}$ exceed the input’s maximum degree L. Relaxing this condition might increase the expressive power of the network but at a significant increase in runtime and memory. We leave the analysis of this trade-off to future work. For the encoder and the decoder to have similar expressive power, we construct them to be symmetric with respect to the latent space [Fig. 1(c)]. Optionally, we apply a linearity at the beginning of the encoder and at the end of the decoder. However, this is required for input data that does not have the same number of channels per degree since the ETP operates channel-wise.

#### f. Tuning the hyperparameters of the training objective

We find it practical to scale the reconstruction loss by a dataset-specific scalar α since the MSE loss varies in average magnitude across datasets. When training H-VAE, we find it beneficial to keep β=0 for a few epochs ($E_{\text{rec}}$) so that the model can learn to perform meaningful reconstructions, and then linearly increasing it to the desired value for $E_{\text{warmup}}$ epochs to add structure to the latent space, an approach first used by Ref. [54].

#### g. Data normalization

As per standard machine learning practice [55], we normalize the data. We do this by dividing each tensor by the average square-root total norm of the training tensors, analogously to the signal norm. This strategy puts the target values on a similar scale as the normalized activations learned by the network, which we speculate to favor gradient flow.

#### h. Pairwise invariant reconstruction loss

To reconstruct a signal within an equivariant model it is desirable to have a *pairwise invariant* reconstruction loss, i.e., a loss ${ℒ}_{\text{rec}}$ such that ${ℒ}_{\text{rec}}(x,y)={ℒ}_{\text{rec}}(D(g)x,D(g)y)$ where D is the representation of the group element g acting on the space that x and y inhabit (e.g., a rotation matrix if x and y are vectors in Euclidean 3D space, or a degree-ℓ Wigner D-matrix if x and y are degree-ℓ vectors). This property is necessary for the model to remain equivariant, i.e., given that the network is agnostic to the transformation of the input under group operation x→D(g)x by producing a similarly transformed output y→D(g)y, we want the reconstruction loss to be agnostic to the same kind of transformation as well.

The MSE loss is pairwise invariant for any degree-ℓ feature on which SO(3) acts via the $\ell^{\prime }$s Wigner D-matrix. Consider two degree-ℓ features $x_{\ell }$ and $y_{\ell }$ acted upon by a Wigner D-matrix $D_{\ell }(g)$ parameterized by rotation g (we drop the g and ℓ indexing for clarity),

(A17)
$$\text{MSE}\left(D_{x},D_{y}\right)={\left(D_{x}-D_{y}\right)}^{T}\left(D_{x}-D_{y}\right)\;={\left(D\left(x-y\right)\right)}^{T}\left(D\left(x-y\right)\right)\;={\left(x-y\right)}^{T}D^{T}D\left(x-y\right)\;={\left(x-y\right)}^{T}\left(x-y\right)\times \text{since}\;\text{Wigner}\;\text{D-matrices}\;\text{are}\;\text{unitary}.\;=\text{MSE}\left(x,y\right)$$

Since the MSE loss is pairwise invariant for every pair of degree-ℓ features, it is thus pairwise invariant for pairs of steerable tensors composed via direct products of steerable features.

#### i. Dependence of the reconstruction loss on feature degree

We observe that features of larger degrees ℓ are harder to reconstruct accurately. Specifically, Fig. S17 within the SM [28] shows that the test reconstruction loss (MSE) increases with degree ℓ for both the MNIST-on-the-sphere and the toy amino acids problems.

#### j. Cosine loss

We use the metric *cosine loss* to measure a model’s reconstruction ability. Cosine loss is a normalized dot product generalized to operate on pairs of steerable tensors (akin to cosine similarity), and modified to be interpreted as a loss (see Sec. A2j for details),

(A18)
$$\text{cosine}\left(x,y\right)=1-\frac{x⊙y}{\sqrt{\left(x⊙x\right)\left(y⊙y\right)}},\;\text{with}\;x⊙y=\underset{\ell^{\prime }}{\sum }{\left(x_{\ell^{\prime }}{⊗}_{cg}y_{\ell^{\prime }}\right)}_{\ell =0.}$$

Importantly, unlike MSE, which depends on the characteristics of the data (e.g., the size of the data tensors), cosine loss is dimensionless and therefore, interpretable and comparable across datasets. A measure with these characteristics is practically useful for evaluating a network because it provides an estimate for how much better the reconstructions can get if the network’s hyperparameters were to be further optimized. For example, looking at the cosine loss in Table S7 within the SM [28], we see that our model trained on Shrec17 (best cosine = 0.130) is not as well optimized as our model trained on MNIST (best cosine = 0.017). Using MSE, the trend is reversed (1.8×10^−3^ vs 6.7×10^−3^), since the scale of MSE depends on the size of the irreps of the data (Fig. S3 within the SM [28]). Nonetheless, as a measure of a model’s reconstruction ability, cosine loss correlates almost perfectly with MSE for a given dataset and network, especially in the mid-to-low reconstruction quality regime (SpearmanR = 0.99, Fig. S3 within the SM [28] and Table S7 within the SM [28]). Crucially, however, as cosine loss ignores the relative norms of data features, it is unable to reconstruct norms and thus, not suitable as a training objective.

##### Proof: Invariance of cosine loss.

The generalized dot product ⊙ from Eq. (A18) is pairwise invariant in the same way that the dot product between two 3D vectors depends only on their relative orientations but not the global orientation of the whole two-vector system. Therefore, the whole cosine loss expression is pairwise invariant, since all of its components are pairwise invariant.

### 3. Using a non equivariant decoder

Winter *et al*. [15] propose to construct group-equivariant autoencoders by using an equivariant encoder that learns an invariant embedding and a group element, and an unconstrained decoder, which uses the invariants alone to reconstruct each datapoint in the “canonical” form, before applying the learned group action in the output space. By contrast, for SO(3) we propose to use an equivariant decoder, whereby the learned group element is fed as input to the decoder. Such “unconstrained decoder” procedure can in principle be merged with our equivariant encoder and Fourierspace approach in two ways. For each, we argue in favor of using our equivariant decoder.

#### (1) Reconstructing the Fourier coefficients of the data.

To apply the learned group element on the decoder’s output, the Wigner D-matrices for the data’s irreps need to be computed from the group element. Then, the Wigner D-matrices can be used to “rotate” the tensor. This has to be done on-the-fly, and it can be done quickly using functions provided in the e3nn package [1] and by smartly vectorizing operations. We implemented this procedure by using a simple multilayer perceptron with SiLU nonlinearities as a decoder. By using e3nn to compute Wigner D-matrices in batches, and by clever construction of tensor multiplications such that runtime scales linearly with $\ell_{max}$ and is constant with regards to number of channels and batch size, we achieve models that run with comparable speed to those using our equivariant decoder, and have comparable performance on MNIST (Table III). Given the empirical similarities we observe, although on a limited use case, we favor the simplicity and elegance of our equivariant decoder. “Simplicity” because we construct the decoder to be symmetric to the encoder, thus endowing it automatically with similar representational power and without the need to tune an architecture made with different base components. Furthermore, we highlight that our method generates intermediate equivariant representations in the decoder, rather than intermediate invariant representations. These intermediate equivariant representations may be of interest to study in and of themselves.

#### (2) Reconstructing the data in real space.

In this case, we do not have to compute Wigner D-matrices on-the-fly, since the learned frame can be used directly in the output space as a rotation matrix. However, since the encoder only sees a truncated Fourier representation of the data, which is by construction lossy, while the loss is computed over fine-grained real-space, this model might be too difficult to train. We suspect this would make the model akin to a denoising autoencoder [56] and it might be interesting to analyze, but that would be beyond the scope of this paper. To avoid the denoising effect, we could learn to reconstruct data in real space after an Inverse Fourier transform (IFT). However, computing the IFT on-the-fly is very expensive and requires a discretization of the input space, to the point of being prohibitive for point clouds. This is not a bottleneck for the forward Fourier transform if the cloud is parameterized by Dirac delta distributions, i.e., for point clouds, as the integral can be computed exactly (Eq. (A9)).

### 4. Implementation details

Without loss of generality, we use real spherical harmonics for implementation of H-(V)AE. We leverage e3nn [1], using their computation of the real spherical harmonics and their Clebsch-Gordan coefficients.

In our code, we offer the option to use the full tensor product instead of the ETP. Specifically, at each block we allow the users to specify whether to compute the tensor product channel-wise or fully connected across channels, and whether to compute using efficient or fully connected degree mixing.

### 5. Experimental details

#### a. Architecture specification

We describe model architectures as follows. We specify the number of blocks B, which is the same for the encoder and the decoder. We specify two lists, (i) DegreesList, which contains the maximum degree $\ell_{max,b}$ of the output of each block b, and (ii) ChannelsList, containing the channel sizes $C_{b}$, of each block b. These lists are in the order as they appear in the encoder, and are reversed for the decoder. When it applies, we specify the number of output channels of the initial linear projection $C_{\text{init}}$. As noted in the main text, we use a fixed formula to determine $\ell_{max,b}$, but we specify it for clarity.

#### b. MNIST on the sphere

##### Model architectures.

For models with invariant latent space size z=16, we use six blocks, DegreesList = [10, 10, 8, 4, 2, 1] and ChannelsList = [16, 16, 16, 16, 16, 16], with a total of 227k parameters.

For models with invariant latent space size z=120, we use six blocks, DegreesList = [10, 10, 8, 4, 2, 1] and ChannelsList = [16, 16, 16, 32, 64, 120], with a total of 453k parameters.

##### Training details.

We keep the learning schedule as similar as possible for all models. We use α=50. We train VAE models for 80 epochs using the Adam optimizer [57] with default parameters, a batch size of 100, and an initial learning rate of 0.001, which we decrease exponentially by one order of magnitude over 25 epochs. We tune β according to the procedure outlined below. During training, we first set β=0 for 25 epochs $\left(E_{rec}=25\right)$ to allow the models to learn to make meaningful reconstructions, and then linearly increase β up to its target value over 35 epochs ($E_{\text{warmup}}=35$). We then the model with lowest validation loss only during the last 20 epochs of training. We instead train AE models for 50 epochs using the “reduce learning rate on plateau” scheme, reducing the learning rate by a factor of 5, if the validation loss does not improve for over one epoch. We utilize the model with the lowest loss on validation data, only after the end of the warmup epochs for the VAE models. Training took ~3.3 minutes per epoch on a single NVIDIA A40 GPU for each model.

##### Tuning the regularization strength β with the help of conditioning.

We select optimal values of β by optimizing the expected quality of the generated samples. We define samples to be of high quality if (i) they can be correctly classified by a classifier trained on real data, and (ii) they are diverse enough. We leverage *conditional* models to generate samples of known digit identity. Specifically, we train an SO(3)-equivariant classifier, with the same architecture as H-(V)AE’s encoder, to classify the digit identity of real data at 97% accuracy. We use this classifier to assess the quality of samples generated by *conditional* H-VAE models trained with varying β. To do so, we sample the conditional models to construct synthetic datasets of 10 000 spherical images, balanced across digit identity. Finally, we compute (i) the accuracy of the classifier on the synthetic datasets, and (ii) the empirical variance of generated samples. We find that there is a trade-off between the two metrics: If β is too low, the generate samples are very diverse but do not resemble the real digits (low classification performance), whereas if β is too high, samples of the same digit start looking more and more alike (i.e., a reduced diversity); see Fig. S9 within the SM [28]. We empirically select β=0.6 and β=2.0 for the z=16 and z=120 models, respectively, based on the trade-off between the above two criteria. We note that this procedure relies on the assumption that the a given regularization strength β has the same effect on the sample quality of the conditional and the unconditional models. We believe this to be likely for the following three reasons: (1) the reconstruction loss is similar for the same values of β (Fig. S6 within the SM [28]); (2) the optimal values of β found using the conditional models approximately optimize the latent space metrics of unconditional models (Fig. S5 within the SM [28]) visual inspection of the conditional vs the unconditional samples across same values of β show similar patterns (Figs. S10 vs S7, S11 vs S8, S12 vs S9 within the SM [28]). We perform this procedure only on the NR/R dataset and use the same selected β to train models on the R/R dataset.

#### c. Shrec17

##### Model architectures.

Both AE and VAE models have z=40, 7 blocks, DegreesList = [14, 14, 14, 8, 4, 2, 1], ChannelsList = [12, 12, 12, 20, 24, 32, 40], $C_{\text{init}}=12$, with a total of 518k parameters.

##### Training details.

We keep the learning schedule as similar as possible for all models. We use α=1000. We train all models for 120 epochs using the Adam optimizer with default parameters, a batch size of 100, and an initial learning rate of 0.0025, which we decrease exponentially by two orders of magnitude over the entire 120 epochs. For VAE models, we use $\beta =0.2,\;E_{\text{rec}}=25$, and $E_{\text{warmup}}=10$. We utilize the model with the lowest loss on validation data, only after the end of the warmup epochs for VAE models. Training took ~11 hours on a single NVIDIA A40 GPU for each model.

#### d. Embedding of amino acid conformations

##### Preprocessing of protein structures.

We sample residues from the set of training structures preprocessed as described in Sec. A5e.

##### Fourier projection.

We set the maximum radial frequency to N=20 as it corresponds to a radial resolution matching the minimum interatomic distances after rescaling the atomic neighborhoods of radius 10.0 Å to fit within a sphere of radius 1.0, necessary for Zernike transform.

The channel composition of the data tensors can be described in a notation—analogous to that used by e3nn but without parity specifications—which specifies the number of channels C for each feature of degree ℓ in single units C×ℓ:44×0+40×1+40×2+36×3+36×4.

##### Model architectures.

All models have z=2,6 blocks, DegreesList = [4, 4, 4, 4, 2, 1], ChannelsList = [60, 40, 24, 16, 16, 8], $C_{\text{init}}=48$, with a total of 495k parameters. We note that the initial projection is necessary since the number of channels differs across feature degrees in the data tensors.

##### Training details.

We keep the learning schedule as similar as possible for all models. We use α=400. We train all models for 80 epochs using the Adam optimizer with default parameters and an initial learning rate of 0.005, which we decrease exponentially by by one order of magnitude over 25 epochs. For VAE models, we use $E_{\text{rec}}=25$ and $E_{\text{warmup}}=10$. We utilize the model with the lowest loss on validation data, only after the end of the warmup epochs for VAE models.

We vary the batch size according to the size of the training and the validation datasets. We use the following (dataset_size-batch_size) pairs: (400–4), (1,000–10), (2,000–20), (5,000–50), (20,000–20). Training took ~45 minutes on a single NVIDIA A40 GPU for each model.

##### Evaluation.

We perform our data ablations by considering training and validation datasets of the following sizes: 400, 1000, 2000, 5000, and 20 000. We keep relative proportions of residue types even in all datasets. We perform the data ablation with H-AE as well as H-VAE models with β=0.025 and 0.1.

We further perform a β ablation using the full (20 000) dataset, over the following choices of β:[0(AE),0.025,0.05,0.1,0.25,0.5].

For robust results, we train three versions of each model and compute averages of quantitative metrics of reconstruction loss and classification accuracy.

For a fair comparison across models with varying amounts of training and validation data, we perform a fivefold cross-validation-like procedure over the 10k test residues, where the classifier is trained over four folds of the test data and evaluated on the fifth one. If validation data is needed for model selection (e.g., for LC), we use 10% of the training data.

#### e. Embedding of protein structure neighborhoods

##### Preprocessing of protein structures.

We model protein neighborhoods extracted from tertiary protein structures from the Protein Data Bank (PDB) [39]. We use ProteinNet’s splittings for training and validation sets to avoid redundancy, e.g., due to similarities in homologous protein domains [41]. Since PDB ids were only provided for the training and validation sets, we used ProteinNet’s training set as both our training and validation set and ProteinNet’s validation set as our testing set. Specifically, we make a [80%, 20%] split in the ProteinNet’s training data to define our training and validation sets. This splitting resulted in 10 957 training structures, 2730 validation structures, and 212 testing structures.

##### Projection details.

We set the maximum radial frequency to N=20 as it corresponds to a radial resolution matching the minimum inter-atomic distances after rescaling the atomic neighborhoods of radius 10.0 Å to fit within a sphere of radius 1.0, necessary for Zernike transform. We vary L and construct models tailored to each one (Table S8 within the SM [28]).

##### Model architectures.

We note that the initial projection ($C_{\text{init}}$) is necessary since the number of channels differs across feature degrees in the data tensors, and the ETP necessitates equal number of channels for all degrees (Sec. A2b and Table S8 within the SM [28]).

##### Training details.

We keep the learning schedule the same for all models. We use α=1000. We train all models for 8 epochs using the Adam optimizer with default parameters, a batch size of 512, and a constant learning rate of 0.001. We utilize the model with the lowest loss on validation data.

#### f. Latent space classification

##### Linear classifier.

We implement the linear classifier as a one-layer fully connected neural network with input size equal to the invariant embedding of size z, and output size equal to the number of desired classes. We use cross entropy loss with logits as training objective, which we minimize for 250 epochs using the Adam optimizer with batch size 100, and initial learning rate of 0.01. We reduce the learning rate by one order of magnitude every time the loss on validation data stops improving for 10 epochs (if validation data is not provided, the training data is used). At evaluation time, we select the class with the highest probability value. We use PyTorch for our implementation.

##### KNN classifier.

We use the sklearn [58] implementation with default parameters. At evaluation time, we select the class with the highest probability value.

#### g. Clustering metrics

##### Purity [34].

We first assign a class to each cluster based on the most prevalent class in it. Purity is computed as the sum of correctly classified items divided by the total number of items. Purity measures classification accuracy, and ranges between 0 (worst) and 1 (best).

##### V-measure [35].

This common clustering metric strikes a balance between favoring homogeneous (high homogeneity score) and complete (high completeness score) clusters. Clusters are defined as homogeneous when all elements in the same cluster belong to the same class (akin to a precision). Clusters are defined as complete when all elements belonging to the same class are put in the same cluster (akin to a recall). The V-measure is computed as the harmonic mean of homogeneity and completeness in a given clustering.

#### h. On the complementary nature of classification accuracy and clustering metrics

The clustering metrics “purity” and “V-measure” and the supervised metric “classification accuracy” characterize different qualities of the latent space, and, while partly correlated, they are complementary to each other.

Both classes of metrics are computed by comparing the ground truth labels to the predicted labels, and they mainly differ by how the predicted labels are assigned; the clustering metrics use an unsupervised clustering algorithm, while the classification metric uses a supervised classification algorithm to do so. As a result, these metrics focus on different features of the latent space. For example, the clustering metrics are largest when the test data naturally forms clusters with all data points of the same label. While this case can result in a high supervised classification accuracy, clustering is not a necessary condition for high classification accuracy. Indeed, the supervised signal could make the predicted labels depend more heavily on a subset of the latent space features, instead of relying on all of them equally, which is what the clustering algorithm naturally does. Therefore, it is reasonable to conclude that having higher clustering metrics and a lower classification accuracy is a sign that class-related information is more evenly distributed across the latent space dimensions. Overall, the complementary aspect of these metrics makes it necessary to use all of them when comparing the performance of different models in each task.

### 6. Protein-Ligand Binding Affinity Prediction

#### a. Data Preprocessing Details

We leverage the H-AE models trained on Protein Neighborhoods (Sec. A5e) to predict the binding affinity between a protein and a ligand, given their structure complex—an important task in structural biology. For this task, we use the data from the “refined-set” of PDBBind [36], containing ~5000 structures. We use the dataset splits provided by ATOM3D [37] to benchmark our predictions. ATOM3D provides two splits based on the maximum sequence similarity between proteins in the training set and the validation/test sets. We use the most challenging split for our benchmarking in which the sequences of the two sets have at most 30% similarity.

For each complex, we first identify residues in the binding pocket, which we define as residues for which the Cα is within 10 Å of any of the ligand’s atoms. We then extract the 10 Å neighborhood for each of the pocket residues that can contain atoms from both the protein and the ligand. We compute the ZFT [Eq. (2)] for each neighborhood with maximum degree L matching the degree used by the H-AE of interest. We then compute residue-level SO(3)-invariant embeddings by running the pretrained H-AE encoder on the neighborhoods surrounding each of the residues in the pocket. We sum over the residue-level embeddings to compute a single pocket-level embedding, which is SE(3) invariant by construction. Each pocket embedding is used as a feature vector within a standard machine learning regression model to predict protein-ligand binding affinity, provided by PDBBind either in the form of a *dissociation constant* $K_{d}$, or an *inhibition constant* $K_{i}$. Due to data limitations, and consistent with other studies on LBA, we do not distinguish between $K_{d}$ and $K_{i}$ and regress over the negative log of either of the constants that is provided by PDBBind; the transformed quantity is closely related to the binding free energy of protein-ligand interactions.

The protein-ligand binding free energy is an extensive quantity, meaning that its magnitude depends on the number of residues in the binding pocket. In other words, a larger protein-ligand complex can establish a stronger binding. The pocket embedding, which we define as the sum of the residue-level embeddings, is a simple quantity that is extensive and SE(3) invariant, and therefore, suitable for regressing over protein-ligand binding free energy. Nonetheless, this map is not unique and other extensive transformations to pool together residue-level embeddings into a pocket-level embedding can be used for this purpose.

It should be noted that in the protein-ligand structural neighborhoods we only include the ligand atoms that are found in proteins and were used in the training of the H-AE models (i.e., C, N, O, and S). About 31% of the ligands contain other kinds of atoms, and since our model does not “see” these atoms, we hypothesize that our predictions are worse in these cases. In fact, for H – AE + R.F. (Table II), the Spearman’s correlation over the ligands containing only protein atoms is higher than for the ligands containing other kinds of atoms by about 0.3 points. Therefore, we expect training H-(V)AE to recognize more atom types—or at least making it aware that some other unspecified non-protein atom is present—would yield even better results; as protein structures are often found in complex with other nonprotein entities, e.g., ligands and ions, there is training data available for constructing such models.

#### b. Machine Learning models used for prediction

##### Linear regression.

We implement a linear regression model as a one-layer fully connected neural network with one output layer. We use MSE loss as training objective, which we minimize for 250 epochs using the Adam optimizer with batch size 32, and initial learning rate of 0.01. We reduce the learning rate by one order of magnitude every time the loss on validation data stops improving for 10 epochs. We use PyTorch for our implementation

##### Random forest regression.

We use the sklearn [58] implementation. We tune hyperparameters via grid search, choosing the combination minimizing RMSD on validation data. We consider the following grid of hyperparameter values:

- max_features: [1.0, 0.333, sqrt]
- min_samples_leaf: [2, 5, 10, 15]
- n_estimators: [32, 64, 100]

#### c. Extended Discussion of Results

Table IV shows benchmarking results on the split of ATOM3D with 30% sequence similarity, extending Table II in the main text with more baselines. Notably, we show the prediction results for the models that only use the SO(3)-invariant (ℓ=0) component of each neighborhood’s Zernike transform (our Zernike inv. baseline), instead of the complete embeddings learned by H-AE. Using H-AE consistently outperforms this baseline, implying that invariant information encoded in higher spherical degrees, and extracted by H-AE, can lead to more expressive models for downstream regression tasks.
