# Supplementary material for: Holographic-(V)AE: An end-to-end SO(3)-equivariant (variational) autoencoder in Fourier space
Source: Phys Rev Res. Author manuscript; Available in PMC 2024 Dec 20. (PMC11661850; doi:10.1103/physrevresearch.6.023006)
Supplement: SI [file NIHMS1999098-supplement-SI.pdf]

Supplementary Information

Holographic-(V)AE: an end-to-end SO(3)-Equivariant (Variational) Autoencoder in Fourier Space
Gian Marco Visani, Michael N. Pun, Arman Angaji, Armita Nourmohammad

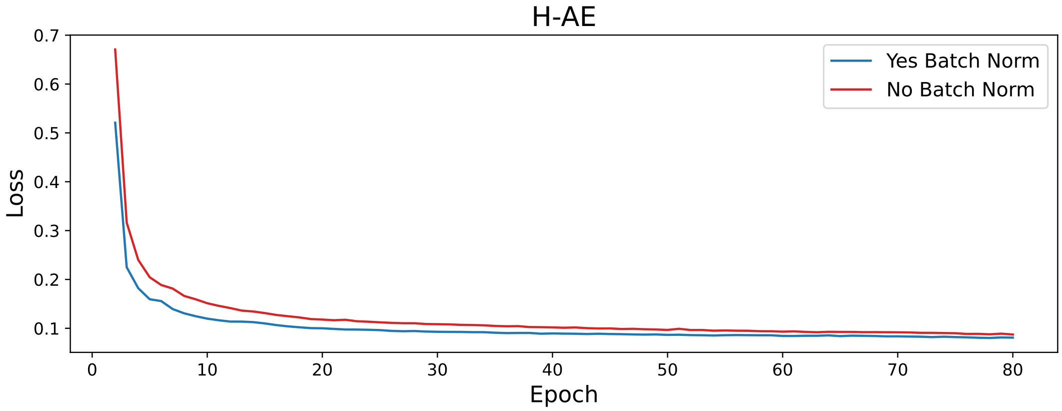

FIG. S1. **Training loss trace of H-AE, with and without Batch Norm, on MNIST-on-the-sphere.** Models were trained with the (NR/R; z = 16; AE) specification. The loss on validation data follows the same trend, but it is not shown for simplicity.

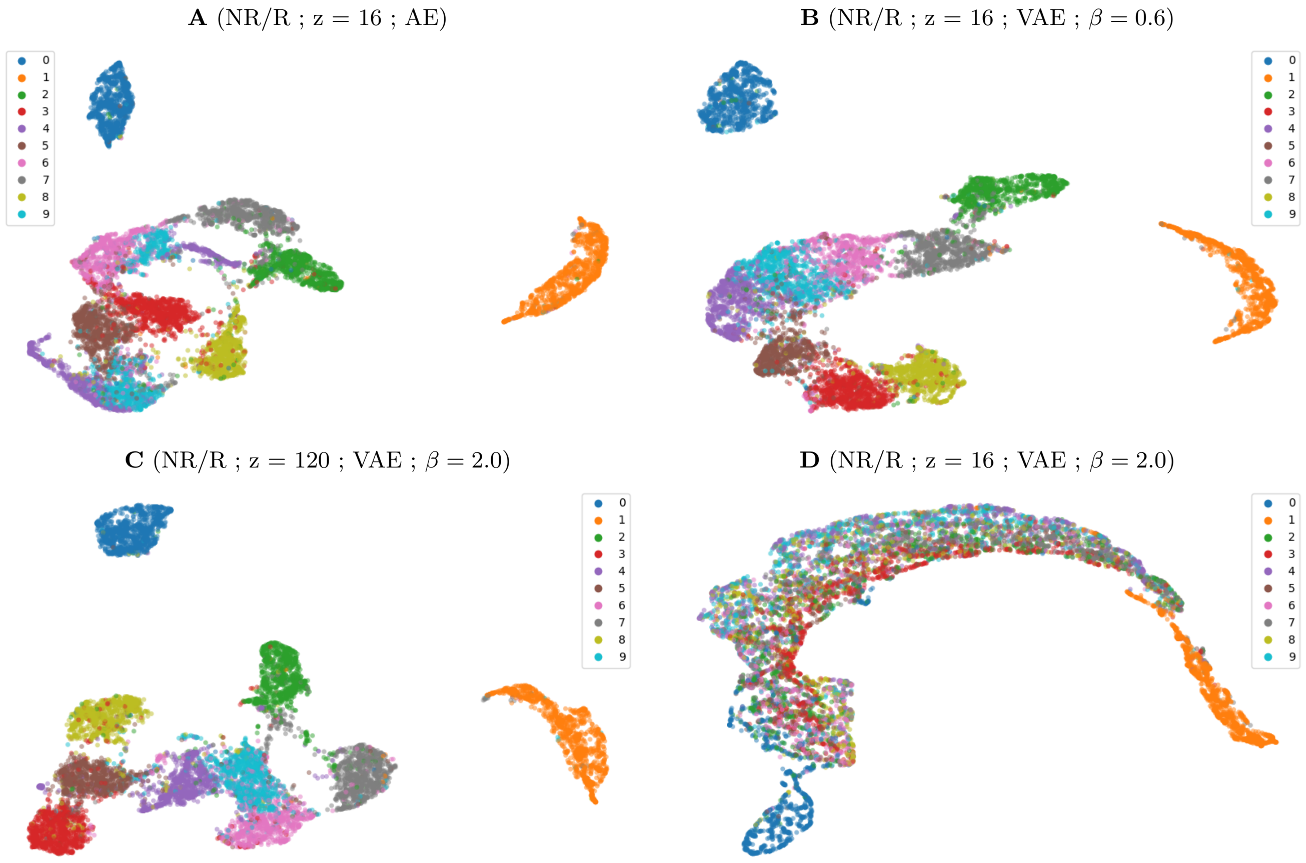

FIG. S2. **UMAP visualization of the invariant latent space learned by selected H-(V)AE models trained on MNIST-on-the-sphere, with varying regularization strengths β.** Variational models with appropriately-tuned regularization strength present the most cohesive clusters based on digit identity (panels B and C). If the regularization is too strong (panel D), the latent space loses most of the digit-based clustering.

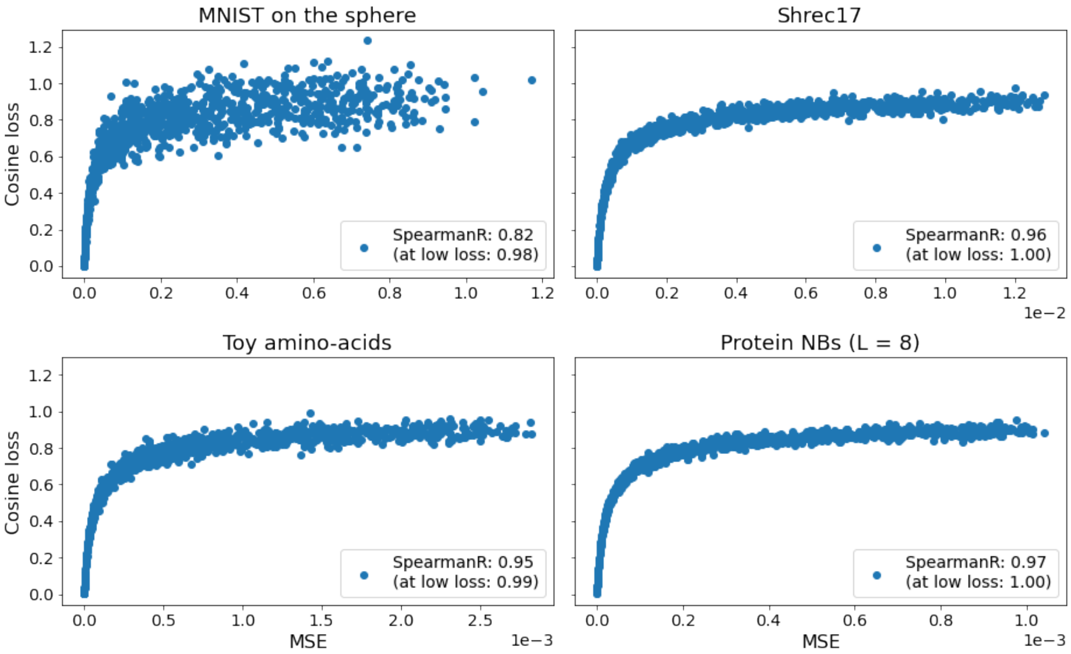

FIG. S3. **Correlation between Cosine loss and MSE values between pairs of random tensors**. For each dataset, we sample a batch of $N = 1000$ tensors with dataset-specific feature degrees and channel sizes, where each coefficient is sampled from a normal distribution. We mimic the normalization step performed in the real experiment and normalize each tensor by the average total norm of the batch. We then generate a "noisy" version of each tensor by adding (normalized) Gaussian noise to each coefficient with standard deviation sampled from a uniform distribution between 0 and some maximum noise level (10 in these plots). This procedure results in N pairs of tensors with varying degrees of similarity between them. We compute the MSE and Cosine loss for all N pairs of tensors and visualize them. The two loss values are well correlated in rank as measured by Spearman Correlation. The correlation is significantly stronger in the regime of reconstruction loss below a Cosine loss of 0.5 (SpearmanR ~ 0.99), a value well above the maximum Cosine loss achieved by H-(V)AE in all our experiments. All the p-values for the Spearman Correlations shown in the plot are significant (< 0.05).

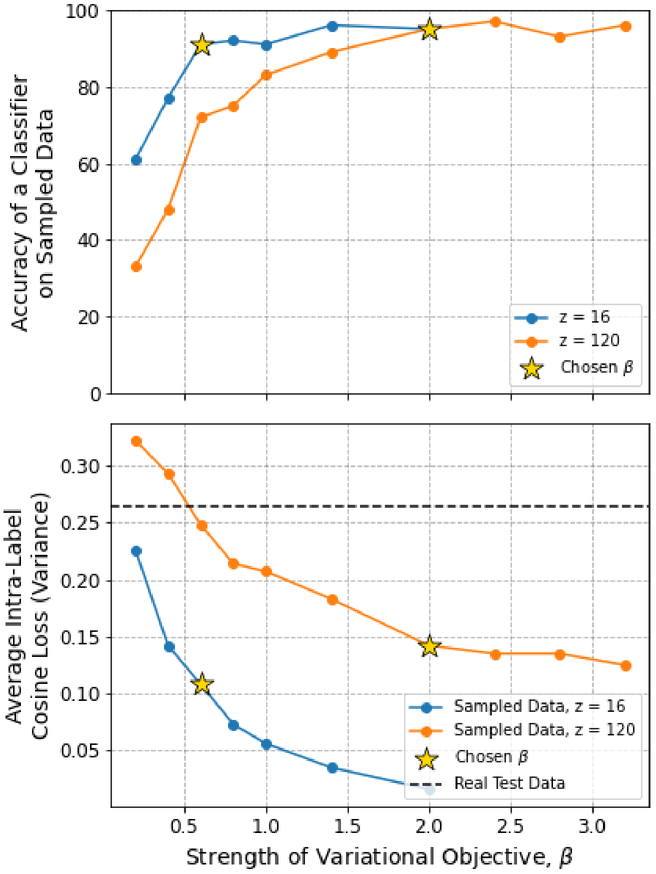

FIG. S4. **Trade-off between classification accuracy (top) and sample diversity (bottom) for the MNIST-on-the-sphere samples generated by H-VAE with digit conditioning, for varying regularization strengths β.** We train conditional H-VAE models with varying values of regularization strength β, as well as an SO(3)-equivariant classifier with high accuracy (97%) on real data. With the conditional H-VAE we generate 10,000 conditional samples evenly distributed across labels. We approximate the samples' quality by the classifier's accuracy (top) and their variance by the intra-label cosine loss across 100,000 samples pairs (bottom); for comparison, the black dotted line indicates the intra-label cosine loss for real data. The plots indicate a clear trade-off between sample quality and diversity. In particular, as β goes above a certain threshold (higher for a larger latent space), the samples start looking alike (see Figure S12 for examples). We chose values of β upon visual inspection and indicate them with a star. We further note that all our models tend to generate samples with lower diversity than real data, indicated by the black dotted line in the bottom plot. This is to be expected since VAEs notoriously tend to "smooth-out" fine details, but the effect is less pronounced with a larger latent space.

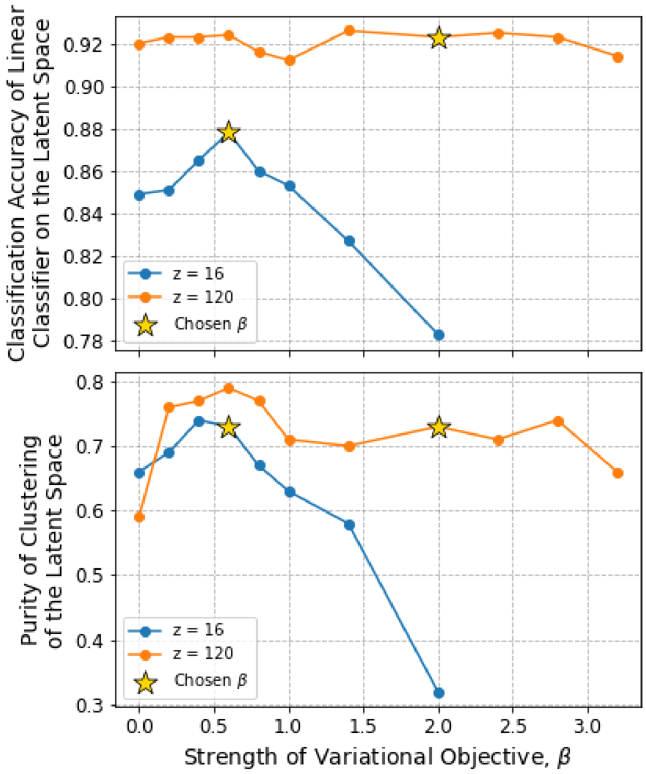

FIG. S5. **Ablation in regularization strength β for MNIST-on-the-sphere test set.** As shown, the chosen values of β (indicated with starts) produce H-VAE models with optimal or near-optimal classification accuracy and purity of clustering. We emphasize that the values of β were chosen according to visual inspection in Figure S4.

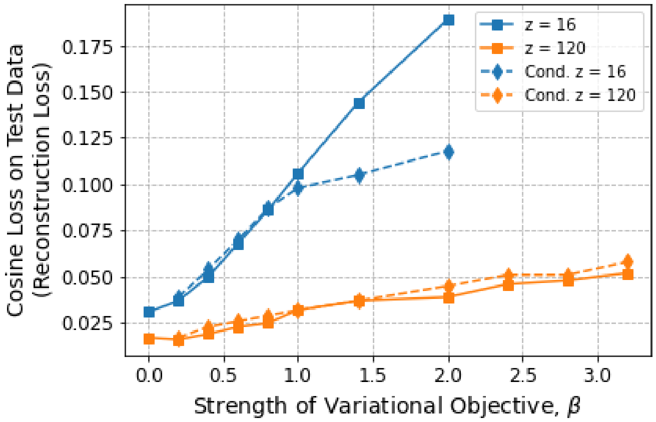

FIG. S6. Reconstruction loss across β for both unconditional and conditional H-VAE models for MNIST-on-the-sphere.

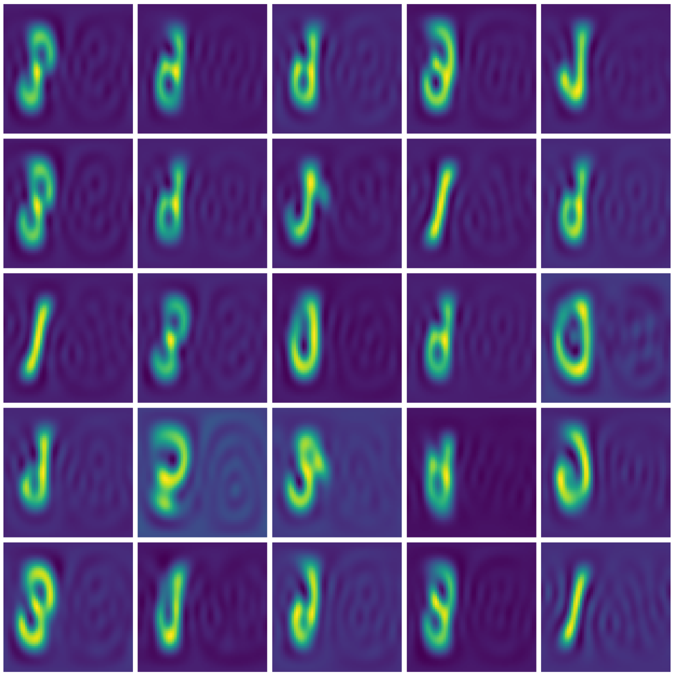

FIG. S7. **Random samples generated by the (NR/R; z = 16; VAE;** $\beta = 0.6$**) MNIST-on-the-sphere model.** We sample invariant latent embeddings from the prior distribution (isotropic Gaussian) and feed them to the decoder alongside the canonical frame to generate tensors. We then compute the inverse spherical Fourier transform to map the generated tensor to images in real space (Eq. A.5). The samples show a wide range of diversity in digit identity and style.

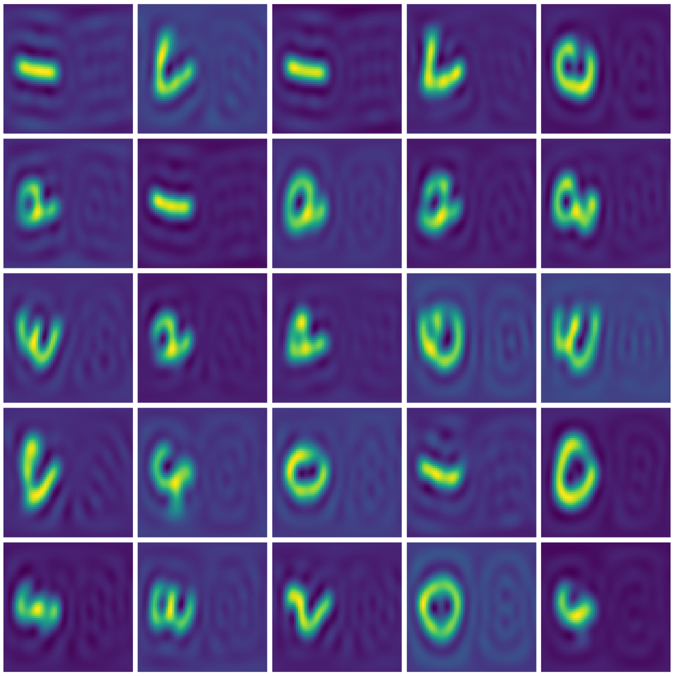

FIG. S8. **Random samples generated by the (NR/R; z = 120; VAE;** $\beta = 2.0$**) MNIST-on-the-sphere model.** Similar to Fig. S7 with different network parameters.

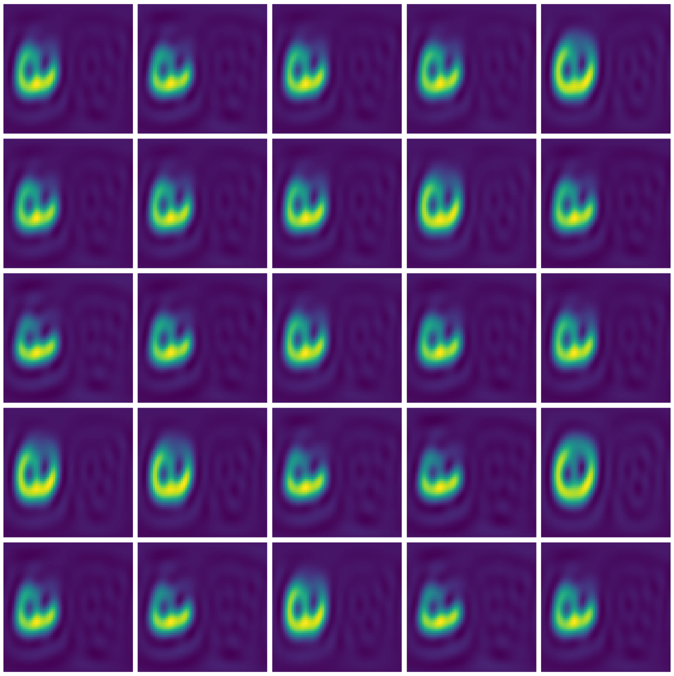

FIG. S9. **Random samples generated by the (NR/R; z = 16; VAE; $\beta = 2.0$) MNIST-on-the-sphere model.** Similar to Fig. S7 with different network parameters. The generated samples look approximately all the same and like a “mush” of all digits, which is a clear sign that the regularization strength β is too high.

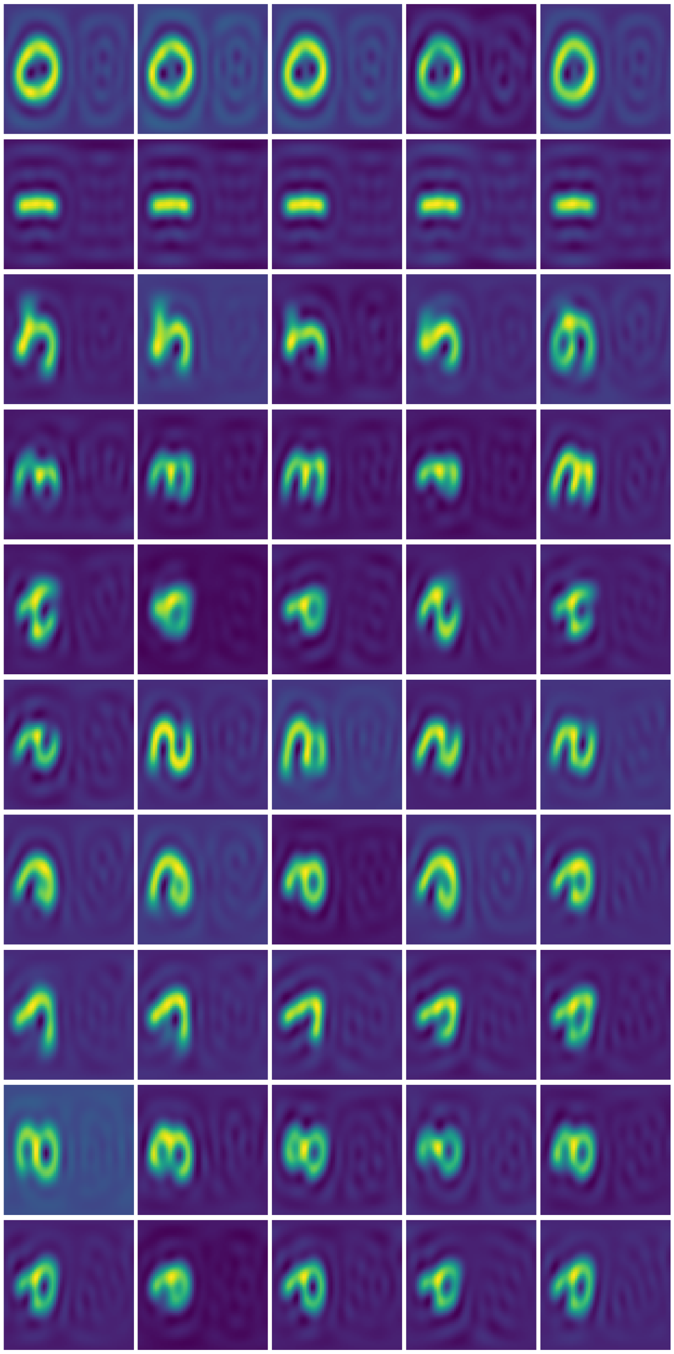

FIG. S10. **Random conditional samples generated by the (NR/R; z = 16; VAE; $\beta = 0.6$; conditional) MNIST-on-the-sphere model.** We sample invariant latent embeddings from the prior distribution (isotropic normal), add digit conditioning, and feed them to the decoder alongside the canonical frame to generate tensors. We then compute the inverse spherical Fourier transform to map the generated tensor to images in real space (Eq. A.5). Samples reflect their conditioning digit and show a wide variety of styles.

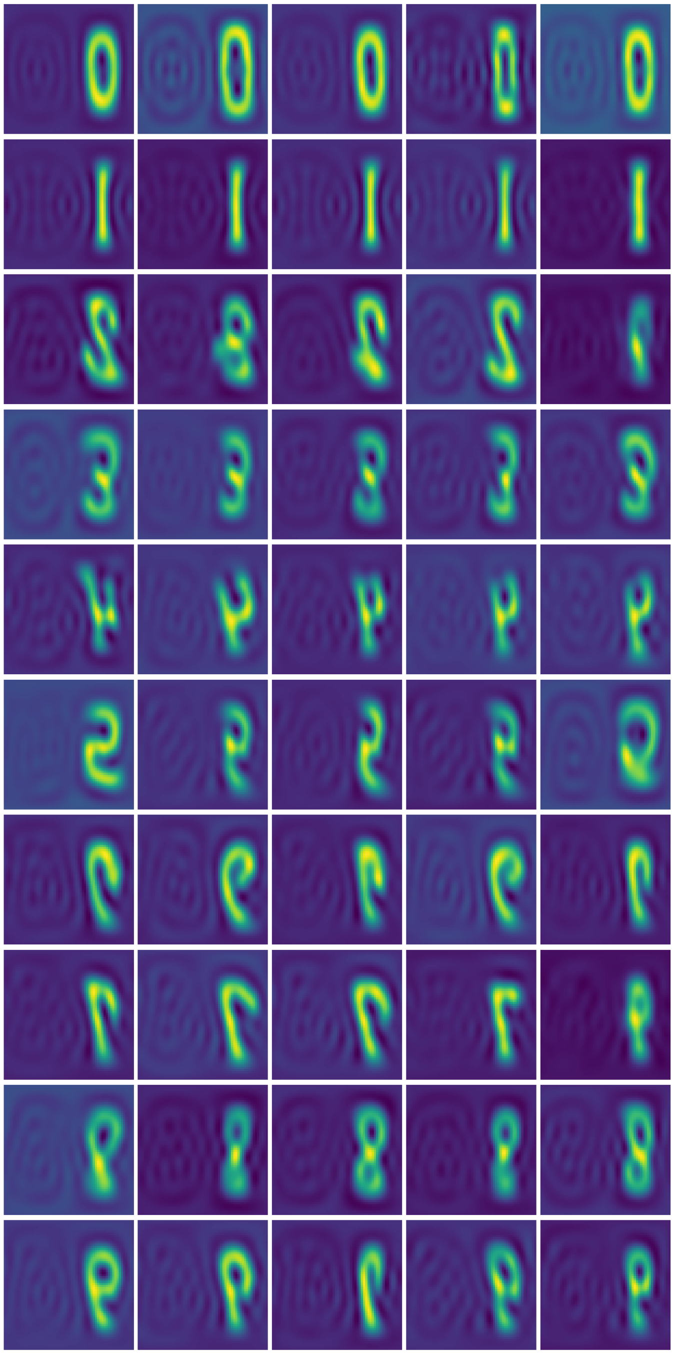

FIG. S11. **Random conditional samples generated by the (NR/R; z = 120; VAE; $\beta = 2.0$; conditional) MNIST-on-the-sphere model.** Similar to Fig. S10 with different network parameters.

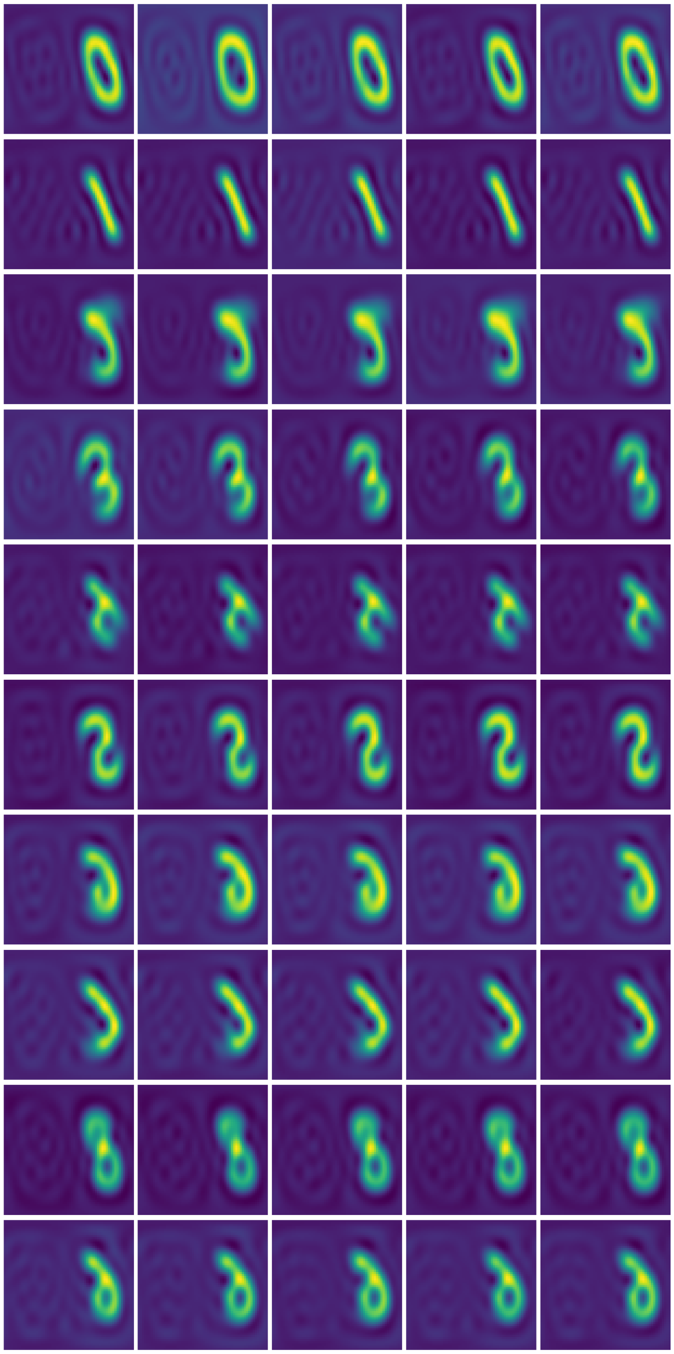

FIG. S12. **Random conditional samples generated by the (NR/R; z = 16; VAE; $\beta = 2.0$; conditional) MNIST-on-the-sphere model.** Similar to Fig. S10 with different network parameters. Samples look the same for each digit label (low diversity), which is a clear sign that the strength of the regularization β is too high.

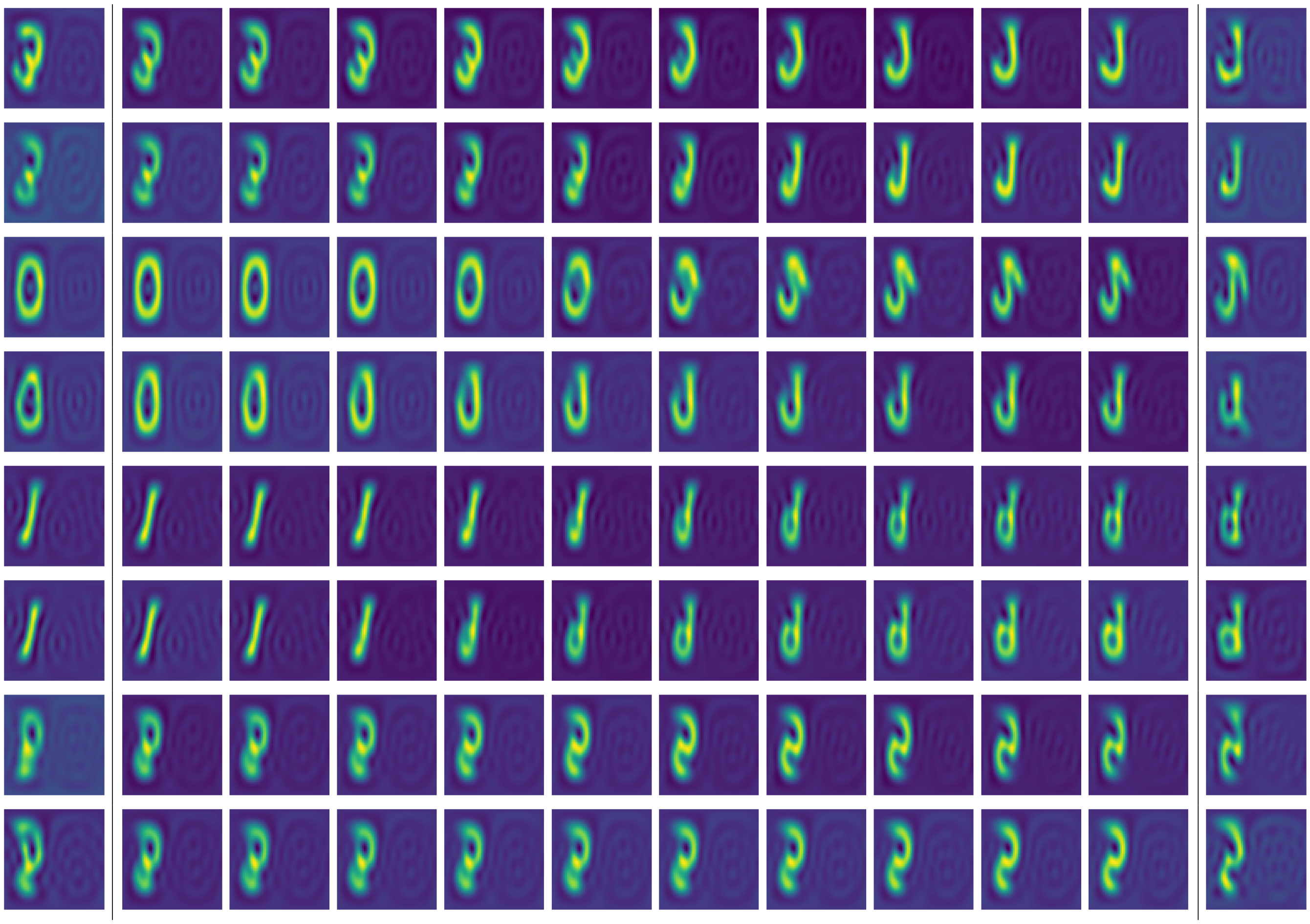

FIG. S13. **Trajectories across the latent space for the (NR/R; z = 16; VAE; $\beta = 0.6$) MNIST-on-the-sphere model.** We compute pairs of invariant latent embeddings using the model's encoder, and linearly interpolate between them through the latent space. We then feed the interpolated embeddings into the decoder, together with the canonical frame, and compute the inverse Spherical Fourier Transform (SFT) (Eq. A.5) to get the image in real space. The left and the right columns show the original images (after forward and inverse SFT) rotated to be placed in the learned canonical frame, whereas the center columns show the interpolated images. We can see that all the trajectories are smooth, respecting the spatial consistency, which is a sign of a well-structured latent space.

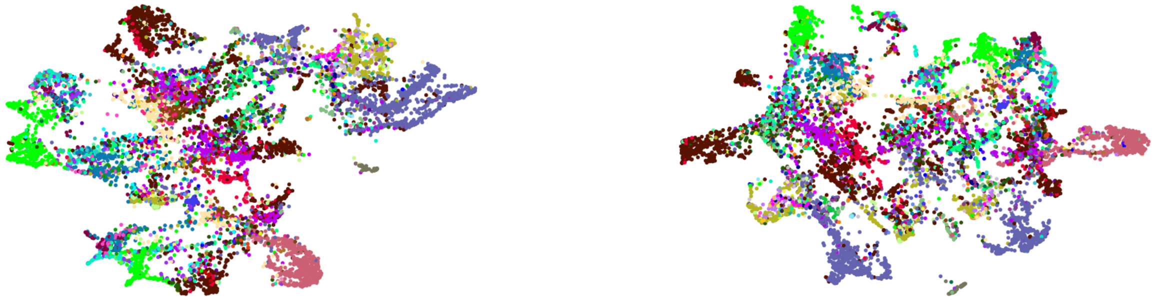

FIG. S14. **2D visualization via UMAP of the invariant latent embeddings of Shrec17 test data learned by H-(V)AE.** Left: H-AE, Right: H-VAE. Points are colored by class (55 classes).

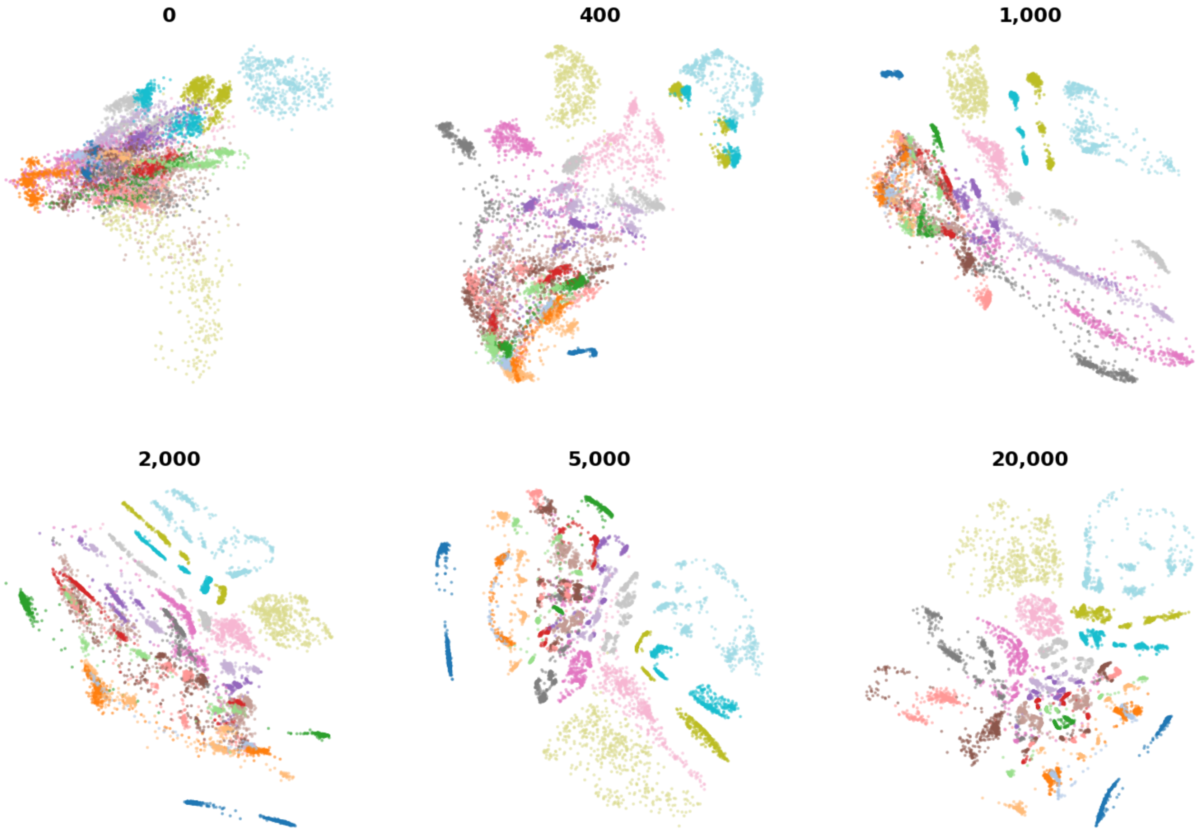

FIG. S15. **Amino acid latent space learned by H-AE.** Visualization of the test data's invariant latent space learned by H-AE trained with varying amounts of the training data. As more training data is added, the separation of clusters containing residues with most similar conformations becomes more distinct. Notably, even with no training data, conformation clusters can be identified.

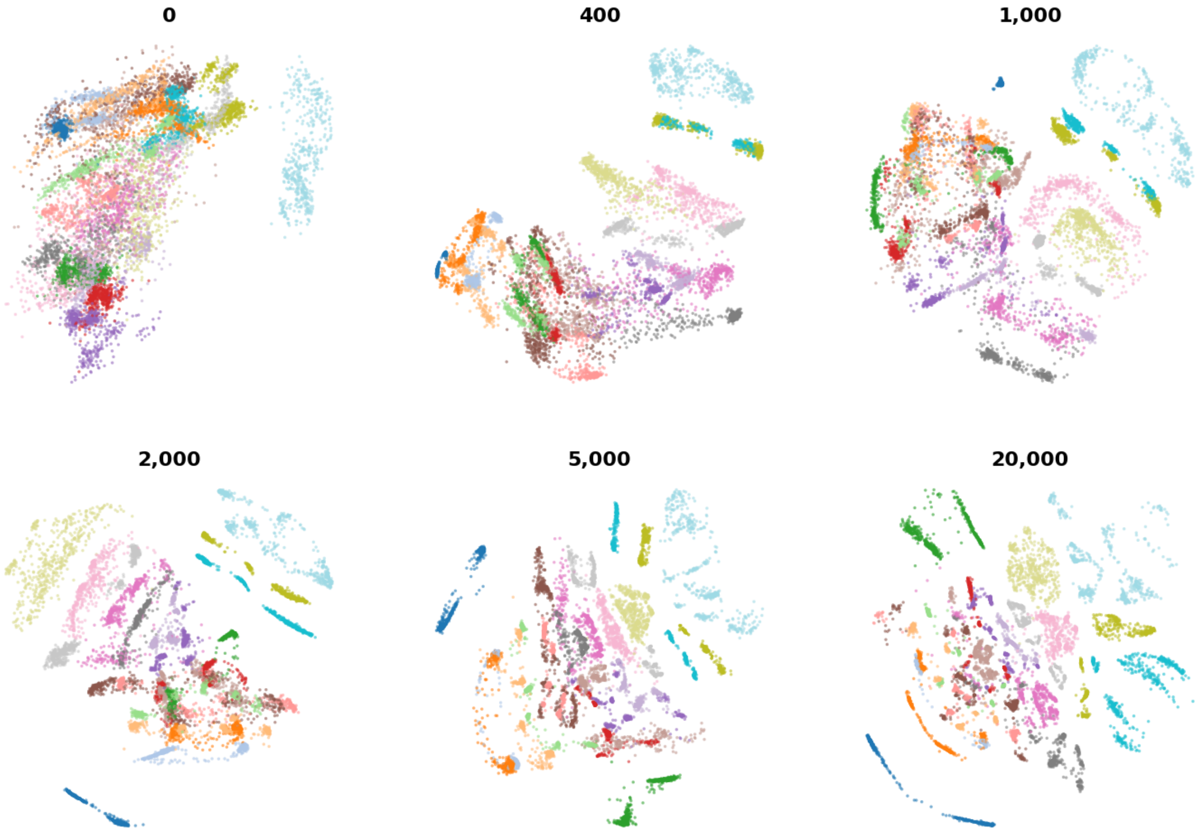

FIG. S16. **Amino acid latent space learned by H-VAE.** Visualization of the test data's invariant latent space learned by H-VAE ($\beta = 0.025$) trained with varying amounts of training data.

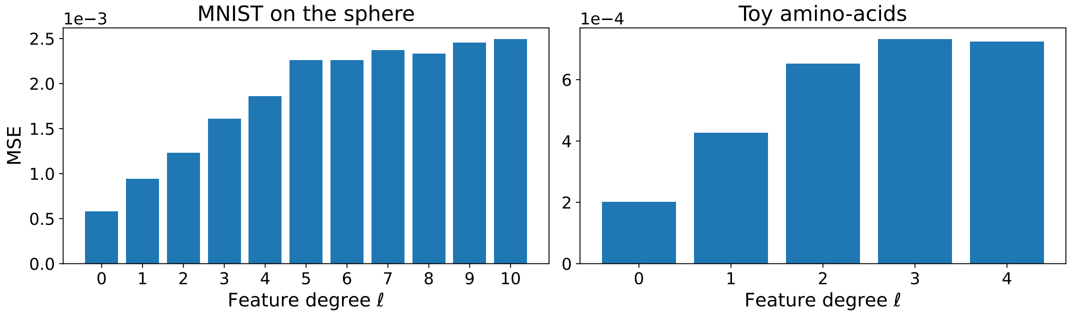

FIG. S17. **Reconstruction loss as a function of feature degree ℓ.** Test reconstruction loss (MSE) of H-VAE split by feature degree ℓ, for the MNIST-on-the-sphere (left) and Toy amino acids dataset (right). In both cases, features of larger degrees are harder to reconstruct accurately. The increase in loss is more steep for smaller degrees.

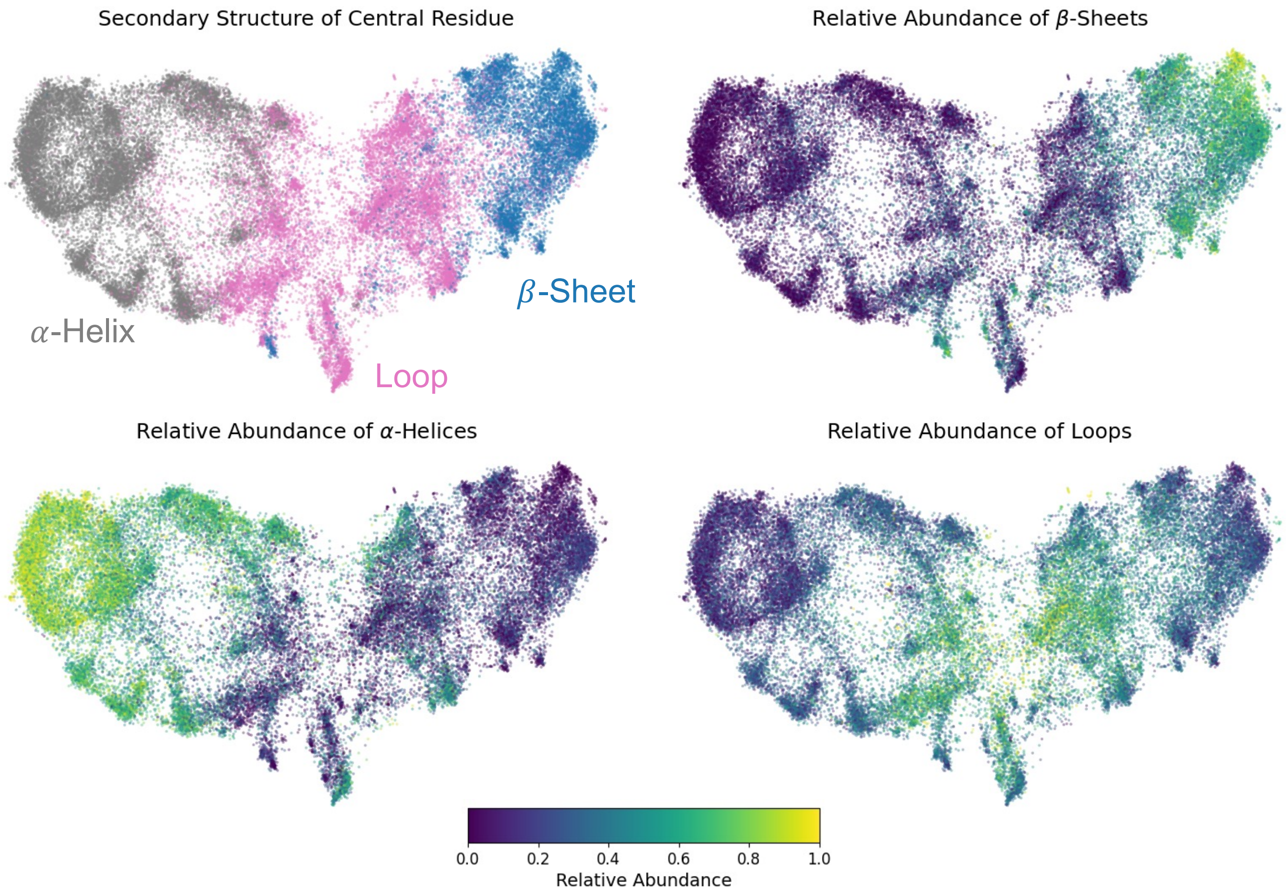

FIG. S18. **Protein structure neighborhoods are well-separated by their secondary structures in the H-AE learned latent space.** Shown are the 2D UMAP visualization of the 128-dimensional invariant latent space learned by H-AE trained on the protein structure neighborhoods with $L = 6$. Within each figure, each point represents a neighborhood. Top left panel: points are colored by secondary structure of the neighborhood's central residue; a logistic regression model trained on the embeddings of 300,000 training neighborhoods is able to recover the central residue's secondary structure on the test set with $\sim 90\%$ accuracy. Other panels: points are colored by the relative abundance of a single secondary structure component (indicated in Figure title), computed as the proportion of atoms within a neighborhood that are assigned to that secondary structure.

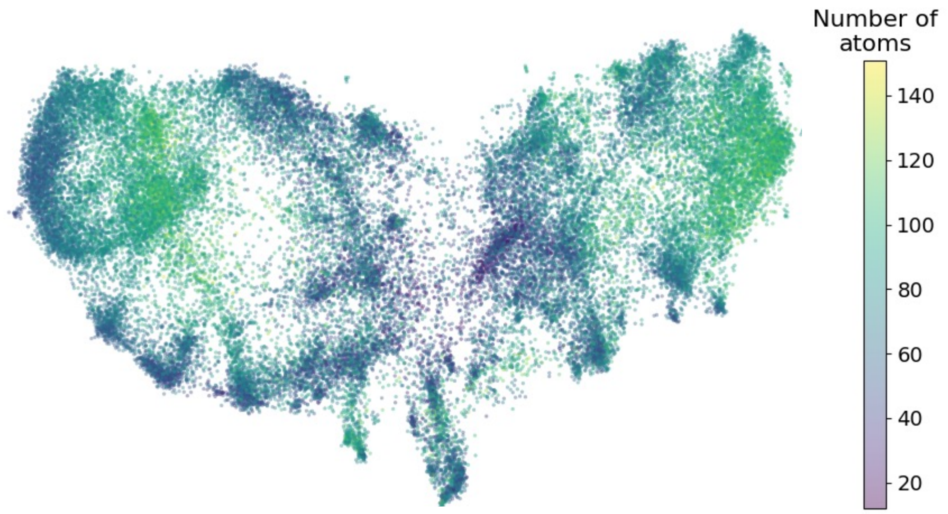

FIG. S19. **The latent space of protein structure neighborhoods annotated by the number of atoms in the neighborhood.** 2D UMAP visualization of the 128-dimensional invariant latent space learned by H-AE trained on the protein structure neighborhoods with $L = 6$. Within each figure, each point represents a neighborhood. Points are colored by the number of atoms in each neighborhood.

**Dataset**	**Method**	L	**z**	**# train**	**Equiv. Error**	**Abs. Value**
MNIST	H-AE NR/R	10	16	-	$(2.3 \pm 2.7)\times 10^{-4}$	$(1.0 \pm 0.9)\times 10^{-1}$
	H-VAE NR/R	10	16	-	$(2.5 \pm 1.9)\times 10^{-4}$	$(1.3 \pm 1.8)\times 10^{-1}$
Shrec17	H-AE	14	40	-	$(1.4 \pm 2.4)\times 10^{-5}$	$(0.4 \pm 1.1)\times 10^{-2}$
	H-VAE	14	40	-	$(1.4 \pm 3.5)\times 10^{-5}$	$(0.4 \pm 2.2)\times 10^{-2}$
Toy Aminoacids	H-AE	4	2	1,000	$(4.7 \pm 3.7)\times 10^{-5}$	$(2.6 \pm 4.1)\times 10^{-2}$
	H-VAE $\beta = 0.025$	4	2	1,000	$(5.4 \pm 3.9)\times 10^{-5}$	$(2.8 \pm 4.0)\times 10^{-2}$
	H-AE	4	2	20,000	$(9.3 \pm 6.4)\times 10^{-5}$	$(3.5 \pm 4.7)\times 10^{-2}$
	H-VAE $\beta = 0.025$	4	2	20,000	$(9.0 \pm 4.7)\times 10^{-5}$	$(3.0 \pm 4.3)\times 10^{-2}$
Protein NBs	H-AE	3	64	-	$(3.3 \pm 4.2)\times 10^{-5}$	$(1.6 \pm 2.1)\times 10^{-2}$
	H-AE	4	64	-	$(0.6 \pm 0.6)\times 10^{-5}$	$(0.6 \pm 2.1)\times 10^{-2}$
	H-AE	6	64	-	$(0.9 \pm 1.0)\times 10^{-5}$	$(0.4 \pm 1.3)\times 10^{-2}$
	H-AE	8	64	-	$(0.7 \pm 1.0)\times 10^{-5}$	$(0.2 \pm 0.9)\times 10^{-2}$
	H-AE	3	128	-	$(1.1 \pm 1.3)\times 10^{-5}$	$(1.0 \pm 3.0)\times 10^{-2}$
	H-AE	4	128	-	$(0.6 \pm 0.7)\times 10^{-5}$	$(0.6 \pm 2.5)\times 10^{-2}$
	H-AE	6	128	-	$(0.1 \pm 0.1)\times 10^{-5}$	$(0.2 \pm 1.7)\times 10^{-2}$
	H-AE	8	128	-	$(0.1 \pm 0.1)\times 10^{-5}$	$(0.1 \pm 1.0)\times 10^{-2}$
	H-AE	3	256	-	$(0.6 \pm 0.4)\times 10^{-5}$	$(0.6 \pm 2.0)\times 10^{-2}$
	H-AE	4	256	-	$(0.4 \pm 0.6)\times 10^{-5}$	$(0.6 \pm 2.2)\times 10^{-2}$
	H-AE	6	256	-	$(0.1 \pm 0.1)\times 10^{-5}$	$(0.2 \pm 1.6)\times 10^{-2}$
	H-AE	8	256	-	$(0.09 \pm 0.07)\times 10^{-5}$	$(0.1 \pm 1.3)\times 10^{-2}$

TABLE S1. **Mean equivariance error for some of our trained H-(V)AE models.** Errors were computed over 2,000 randomly sampled spherical tensors, each with a randomly sampled rotation. Standard deviation is shown alongside the mean. We also show the mean and standard deviation of the absolute value of the output coefficients, to enable contextualization of the measured equivariance error. The equivariance error due to numerical error (absolute difference in coefficients by rotating input vs. output tensor) is consistently three orders of magnitude lower than the typical absolute value of the coefficients, indicating that equivariance is preserved. The same trend occurs for *untrained* models (not shown here for simplicity).

Dataset	Method	L	z	TP-type	C_{init}	ChannelsList	DegreesList	# Params	Speed	MSE	Cosine
MNIST	H-AE	10	16	ETP	None	[16,16,16,16,16,16]	[10,10,8,4,2,1]	227k	**1.0**	1.2×10^{-3}	0.031
	H-AE	10	16	Full-TP	None	[7,6,6,6,16]	[10,8,4,2,1]	229k	1.1	9.8×10^{-4}	0.025
	H-AE	10	16	Full-TP	None	[5,5,5,5,7,16]	[10,10,8,4,2,1]	227k	1.7	$\mathbf{8.9 \times 10^{-4}}$	**0.024**
Shrec17	H-AE	14	40	ETP	12	[12,12,12,20,24,32,40]	[14,14,14,8,4,2,1]	518k	1.0	$\mathbf{1.8 \times 10^{-4}}$	**0.130**
	H-AE	14	40	Full-TP	None	[5,5,5,8,40]	[14,8,4,2,1]	518k	**0.9**	1.9×10^{-4}	0.137
	H-AE	14	40	Full-TP	None	[4,3,3,6,6,6,40]	[14,14,14,8,4,2,1]	513k	1.9	2.0×10^{-4}	0.142
Protein NBs	H-AE	6	64	ETP	44	[50,50,50,64,64,64]	[6,6,6,4,2,1]	1.6M	**1.0**	$\mathbf{5.4 \times 10^{-4}}$	**0.219**
	H-AE	6	64	Full-TP	None	[8,8,12,36]	[6,4,2,1]	1.6M	2.1	6.7×10^{-4}	0.286
	H-AE	8	64	ETP	44	[50,50,50,64,64,64]	[6,6,6,4,2,1]	2.4M	**1.0**	$\mathbf{6.3 \times 10^{-4}}$	**0.287**
	H-AE	8	64	Full-TP	None	[8,8,12,36]	[6,4,2,1]	2.6M	3.3	7.6×10^{-4}	0.371

TABLE S2. **Training speed and reconstruction ablations of H-(V)AE models with different Tensor Product rules.** To make comparison fair, models were trained using the same training hyperparameters as described in Section A 5, and all models were constructed to have comparable number of parameters. Speed was computed as training time and divided by the time of the model using ETP within each dataset. Models with the ETP usually generate better reconstructions and are usually the fastest. The speed and performance gains of the ETP are most apparent on the Protein Neighborhoods task, where we also note that, as the angular resolution of the data (L) is increased from 6 to 8, the relative speed gain of the ETP over the Full-TP is significantly accentuated (from 2.1x to 3.3x).

Type	Method	z	bw	LC Acc.	KNN Acc.
Unsupervised	H-AE NR/R	120	30	0.920	0.920
	H-AE R/R	120	30	0.916	**0.923**
	H-AE NR/R	16	30	0.850	0.902
	H-AE R/R	16	30	0.844	0.901
	H-VAE NR/R	120	30	**0.923**	0.905
	H-VAE R/R	120	30	**0.923**	0.914
	H-VAE NR/R	16	30	0.878	0.897
	H-VAE R/R	16	30	0.855	0.880

TABLE S3. **Evaluation of network performances for MNIST-on-the-sphere using a K-Nearest Neighbors (KNN) classifier instead of linear classifier in the latent space.** Results are significantly better than when using a linear classifier for models with smaller ($z = 16$) latent space, comparable or worse for the other models. Interestingly, VAE models have worse KNN classification performance than AE models, reversing the trend seen with Linear Classification.

Type	Method	z	bw	Class. Acc.	P@N	R@N	F1@N	mAP	NDCG
Unsupervised + LC	H-AE	40	90	0.654	0.548	0.569	0.545	0.500	0.597
	H-VAE	40	90	0.631	0.512	0.537	0.512	0.463	0.568
Unsupervised + KNN	H-AE	40	90	**0.672**	**0.560**	**0.572**	**0.555**	**0.501**	**0.599**
	H-VAE	40	90	0.658	0.541	0.558	0.539	0.487	0.591

TABLE S4. **Evaluation of network performances for Shrec17 using a KNN classifier instead of linear classifier in the latent space.** Results are better than when using a linear classifier.

	H-AE				H-VAE ($\beta = 0.025$)				H-VAE ($\beta = 0.1$)			
# train	MSE	Cosine loss	LC Acc.	KNN Acc.	MSE	Cosine loss	LC Acc.	KNN Acc.	MSE	Cosine loss	LC Acc.	KNN Acc.
0	1.3×10^{-2}	1.015	0.409	0.629	1.5×10^{-2}	0.981	0.424	0.656	1.5×10^{-2}	0.981	0.424	0.656
400	9.4×10^{-4}	0.153	0.586	0.842	9.8×10^{-4}	0.160	0.616	0.848	1.0×10^{-3}	0.163	0.558	0.780
1,000	5.9×10^{-4}	0.099	0.583	0.856	6.3×10^{-4}	0.101	0.569	0.854	6.9×10^{-4}	0.113	0.564	0.844
2,000	4.5×10^{-4}	0.073	0.560	0.900	4.9×10^{-4}	0.081	0.554	0.905	5.5×10^{-4}	0.092	0.593	0.890
5,000	3.3×10^{-4}	0.053	0.629	0.940	3.3×10^{-4}	0.053	0.638	0.961	4.3×10^{-4}	0.072	0.588	0.921
20,000	2.2×10^{-4}	0.034	0.578	0.972	2.4×10^{-4}	0.037	0.667	0.971	2.9×10^{-4}	0.047	0.662	0.966

TABLE S5. **Quantitative data ablation results on the toy amino acids dataset.** A random-guessing classifier has an expected accuracy of 0.050.

β	MSE	Cosine	LC Acc.	KNN Acc.
0 (AE)	2.2×10^{-4}	0.034	0.580	0.972
0.025	2.4×10^{-4}	0.037	0.666	0.971
0.05	2.5×10^{-4}	0.039	0.669	0.968
0.1	2.9×10^{-4}	0.047	0.661	0.966
0.25	6.8×10^{-4}	0.132	0.597	0.854
0.5	1.1×10^{-3}	0.203	0.467	0.722

TABLE S6. **Quantitative data ablation results for variational objective on the toy amino acids dataset.** Models were trained on the full dataset (# train = 20,000).

Dataset	Method	L	z	bw	# Params	MSE	Cosine
MNIST	H-AE NR/R	10	120	30	453k	6.5×10^{-4}	0.017
	H-AE R/R	10	120	30	453k	5.8×10^{-4}	0.015
	H-AE NR/R	10	16	30	227k	1.2×10^{-3}	0.031
	H-AE R/R	10	16	30	227k	1.2×10^{-3}	0.030
	H-VAE NR/R	10	120	30	453k	1.5×10^{-3}	0.039
	H-VAE R/R	10	120	30	453k	1.6×10^{-3}	0.041
	H-VAE NR/R	10	16	30	227k	2.8×10^{-3}	0.068
	H-VAE R/R	10	16	30	227k	2.7×10^{-3}	0.067
Shrec17	H-AE	14	40	90	518k	1.8×10^{-4}	0.130
	H-VAE	14	40	90	518k	2.2×10^{-4}	0.151
Protein NBs	H-AE	3	64	-	631k	3.3×10^{-4}	0.084
	H-AE	4	64	-	950k	3.9×10^{-4}	0.125
	H-AE	6	64	-	1.6M	5.4×10^{-4}	0.219
	H-AE	8	64	-	2.4M	6.3×10^{-4}	0.287
	H-AE	3	128	-	1.2M	2.1×10^{-4}	0.053
	H-AE	4	128	-	1.7M	2.8×10^{-4}	0.087
	H-AE	6	128	-	2.6M	4.0×10^{-4}	0.156
	H-AE	8	128	-	3.8M	4.9×10^{-4}	0.213
	H-AE	3	256	-	2.8M	1.0×10^{-4}	0.025
	H-AE	4	256	-	3.8M	1.9×10^{-4}	0.060
	H-AE	6	256	-	4.4M	2.7×10^{-4}	0.102
	H-AE	8	256	-	7.9M	3.5×10^{-4}	0.148

TABLE S7. **Test MSE and Cosine loss for H-(V)AE models trained on MNIST, Shrec17 and Protein Neighborhoods.** MSE and Cosine values are strongly correlated within datasets but not across datasets.

z	L	Tensor size	$\mathbf{C_{init}}$	ChannelsList	DegreesList	# Params	Training Speed (hours)
64	3	616	44	[50,50,50,64,64,64]	[3,3,3,3,2,1]	631k	3.0
64	4	940	44	[50,50,50,64,64,64]	[4,4,4,4,2,1]	950k	3.8
64	6	1708	44	[50,50,50,64,64,64]	[6,6,6,4,2,1]	1.6M	5.9
64	8	2604	44	[50,50,50,64,64,64]	[8,8,8,4,2,1]	2.4M	9.3
128	3	616	44	[60,60,60,90,128,128]	[3,3,3,3,2,1]	1.2M	3.8
128	4	940	44	[60,60,60,90,128,128]	[4,4,4,4,2,1]	1.7M	4.4
128	6	1708	44	[60,60,60,90,128,128]	[6,6,6,4,2,1]	2.6M	6.0
128	8	2604	44	[60,60,60,90,128,128]	[8,8,8,4,2,1]	3.8M	10.1
256	3	616	44	[90,90,90,120,200,256]	[3,3,3,3,2,1]	2.8M	4.9
256	4	940	44	[90,90,90,120,200,256]	[4,4,4,4,2,1]	3.8M	5.6
256	6	1708	44	[90,90,90,120,200,256]	[6,6,6,4,2,1]	4.4M	6.0
256	8	2604	44	[90,90,90,120,200,256]	[8,8,8,4,2,1]	7.9M	14.5

TABLE S8. **Model architecture hyperparameters and training time for each H-AE trained on protein neighborhoods.**
